# KRBP72 facilitates ATPase-dependent editing progression through a structural roadblock in mitochondrial A6 mRNA

**DOI:** 10.1093/nar/gkae1153

**Published:** 2024-12-03

**Authors:** Ashutosh P Dubey, Brianna L Tylec, Soon Yi, Frank A Tedeschi, Joseph T Smith, Laurie K Read

**Affiliations:** Department of Microbiology and Immunology, University at Buffalo Jacobs School of Medicine and Biomedical Sciences, 955 Main Street, Buffalo, NY 14203, USA; Department of Microbiology and Immunology, University at Buffalo Jacobs School of Medicine and Biomedical Sciences, 955 Main Street, Buffalo, NY 14203, USA; Center for RNA Science and Therapeutics, School of Medicine, Case Western Reserve University, 2109 Adelbert Rd., Cleveland, OH 44106, USA; Department of Biochemistry, School of Medicine, Case Western Reserve University, 2109 Adelbert Rd., Cleveland, OH 44106, USA; Center for RNA Science and Therapeutics, School of Medicine, Case Western Reserve University, 2109 Adelbert Rd., Cleveland, OH 44106, USA; Department of Biochemistry, School of Medicine, Case Western Reserve University, 2109 Adelbert Rd., Cleveland, OH 44106, USA; Department of Microbiology and Immunology, University at Buffalo Jacobs School of Medicine and Biomedical Sciences, 955 Main Street, Buffalo, NY 14203, USA; Department of Microbiology and Immunology, University at Buffalo Jacobs School of Medicine and Biomedical Sciences, 955 Main Street, Buffalo, NY 14203, USA

## Abstract

Uridine insertion/deletion editing of mitochondrial messenger RNAs (mRNAs) in kinetoplastids entails the coordinated action of three complexes. RNA Editing Catalytic Complexes (RECCs) catalyze the enzymatic reactions, while the RNA Editing Substrate Binding Complex (RESC) and RNA Editing Helicase 2 Complex (REH2C) coordinate interactions between RECCs, mRNAs and hundreds of guide RNAs that direct edited sequences. Additionally, numerous auxiliary factors are required for productive editing of specific mRNAs. Here, we elucidate the role of KRBP72, an editing auxiliary factor of the ABC adenosine triphosphatase (ATPase) family that exhibits RNA-binding activity. In procyclic form *Trypanosoma brucei*, KRBP72 knockdown leads to a pause in editing at the base of a predicted stem loop structure in adenosine triphosphate synthase subunit 6 (A6) mRNA. Enhanced cross-linking and affinity purification revealed KRBP72 binding sites both within and upstream of this stem loop. KRBP72 ATPase activity is essential for its A6 mRNA editing function; however, its RNA-binding activity is dispensable. KRBP72 interacts with most RESC proteins in an RNase-sensitive manner. By contrast, RESC12A associates with KRBP72 in an RNase-insensitive fashion, and RESC12A promotes KRBP72’s interaction with RNA. Hence, KRBP72 ATPase activity facilitates progression of editing through a challenging secondary structure, highlighting this protein's crucial role in A6 mRNA editing.

## Graphical Abstract

**Graphical Abstract:**
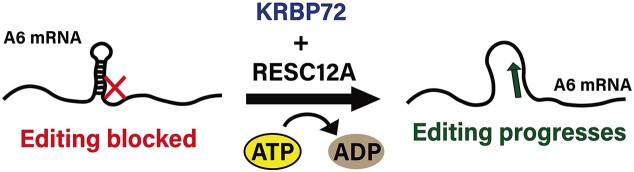

## Introduction

Uridine insertion/deletion (U-indel) RNA editing entails extensive post-transcriptional modification of mitochondrial messenger RNAs (mRNAs) and is unique to kinetoplastid organisms, such as the human and livestock parasite, *Trypanosoma brucei*. During this process, uridine residues are added to and, less frequently, deleted from mRNAs to correct frameshifts and create entire open reading frames, thereby generating translatable mRNAs and sometimes doubling the size of a transcript. U-indel editing is directed by mitochondrially encoded guide RNAs (gRNAs) (1,2). For the nine *T. brucei* mitochondrial mRNAs that are edited throughout their lengths, termed pan-edited, dozens of gRNAs act sequentially to facilitate correct editing of one mRNA. Editing occurs in a general 3′ to 5′ direction along the mRNA because the editing directed by a given gRNA produces the edited sequence required for anchoring the subsequent gRNA. Most edited mRNA populations primarily consist of partially edited transcripts undergoing the editing process, as verified by numerous high-throughput studies (3–6). In addition, while editing proceeds generally from 3′ to 5′, partially edited mRNAs often contain ‘junctions’ at the leading edge of editing. Many of these junctions, which are not pre-edited or canonically edited, are likely regions of ongoing editing (3,5,7).

Over 50 proteins with a role in U-indel editing have been described, the majority of which are components of three complexes that together comprise the editing holoenzyme (8). Of these, 21 have been ascribed to the RNA Editing Catalytic Complexes (RECCs), three related complexes that contain enzymes for endonuclease cleavage, U insertion/deletion and RNA ligation (9–11). RECCs edit multiple mRNA sites non-processively during RNA editing (12). An additional 19 proteins are considered components of the RNA Editing Substrate binding Complex (RESC; previously MRB1 complex) that serves as the platform for RNA editing and facilitates interactions between RECC, mRNA and gRNA. RESC itself comprises distinct modules, and recent studies have identified RESC components that regulate RESC organization, thereby promoting editing initiation and/or 3′ to 5′ editing progression (5,13–16). A recent cryo-electron microscopy (cryo-EM) study supports the dynamic nature of RESC, as it identified three RESC forms that likely exchange proteins and undergo rearrangement during editing (17). The third component of the editing holoenzyme, REH2C, consists of three different proteins, including the KREH2 adenosine triphosphate (ATP)-dependent RNA helicase, which influences the accuracy of RNA editing in a site-specific and substrate-specific manner for RESC-associated transcripts (6,18) and which exerts developmental control over programmed non-canonical editing events (19,20).

Several additional proteins known as auxiliary factors associate with RESC, often in an RNase-sensitive manner (8). These proteins tend to impact the editing of specific mRNAs, although their mechanisms of action are not well understood (7,21–26). For example, RNA interference (RNAi)-mediated depletion of either KMRP1/2 or KRBP16 results in the loss of edited apocytochrome b (CYb) mRNA in procyclic form (PCF) *T. brucei* (7,21,22). Both proteins possess RNA annealing activities that may contribute to their abilities to facilitate CYb mRNA editing (27,28). However, genetic studies demonstrated that they function through distinct mechanisms, and KMRP1/2 likely functions at least partially through modulation of edited CYb mRNA stability (7,29). KRGG1 is an RNA-binding protein for which conditional knock out in bloodstream form (BSF) *T. brucei* established its essentiality in the 3′ to 5′ progression of adenosine triphosphatase (ATPase) subunit 6 (A6) mRNA editing through a specific region of the transcript (26,30). Interestingly, KRGG1 also repressed editing of the NADH dehydrogenase subunit 8 mRNA. Mutational studies showed that ARM/HEAT repeats in KRGG1 are critical to its association with RESC and its ability to support normal growth and edited A6 mRNA levels. Studies in PCF *T. brucei* demonstrated that the DEAD box RNA helicase, KREH1, is essential for editing of A6 mRNA, and overexpression studies suggest that it can impact editing of additional transcripts in a manner redundant with an unknown factor (31). KREH1 likely acts by modulation of RNA structure, particularly with regard to initiator gRNAs. Another auxiliary factor, which apparently lacks RNA-binding activity, is the homotrimeric P22 (25). P22 dramatically and specifically affects editing of the cytochrome oxidase subunit II (COII) mRNA in PCF, and is essential for the developmental induction of COII mRNA editing (25,32). Thus, U-indel mRNA editing auxiliary factors appear to utilize diverse activities to promote editing of distinct transcripts.

In this manuscript, we investigate the mechanism of action of the auxiliary factor, KRBP72 (Tb927.3.1590; previously MRB1590) (23). KRBP72 was first described in association with the RESC component, RESC1, by mass spectrometry of both immunoprecipitates and tandem affinity purifications (TAP) (33). Subsequently, its association with RESC8, RESC12, RESC12A and KREH2 were reported (34–36), further suggesting a function in U-indel editing. Indeed, Shaw *et al.* demonstrated the involvement of KRBP72 specifically in the editing of the mitochondrial A6 transcript (23). In this same study, the KRBP72 crystal structure was solved revealing that the protein is dimeric, featuring a central ABC ATPase fold embedded between unique N- and C-terminal regions (23). The N-terminal domains collectively form a basic pore implicated in RNA binding, as supported by biochemical studies. Structural investigations captured distinct KRBP72 conformations, showcasing a dynamic RNA-binding pore that alternates between closed and open states; the latter is capable of accommodating RNA (23). The existence of ATPase activity was also demonstrated. Here, we combine transcriptomic and biochemical studies to elucidate KRBP72 function in U-indel editing in PCF *T. brucei*. We identify a specific roadblock, comprising a putative stable stem loop structure in A6 mRNA, that requires KRBP72 for its transversal and show that the protein binds pre-edited A6 sequence near this site. KRBP72 ATPase activity is needed for editing to progress past this site, while its own RNA-binding activity is largely dispensable. Instead, an RNase-insensitive interaction between KRBP72 and the RNA-binding protein, RESC12A, is necessary for the KRBP72-A6 mRNA interaction. Together, our studies lead to a model for the action of the auxiliary factor, KRBP72, in U-indel editing.

## Material and methods

### *T. brucei* cell line generation

**Figure 1.**
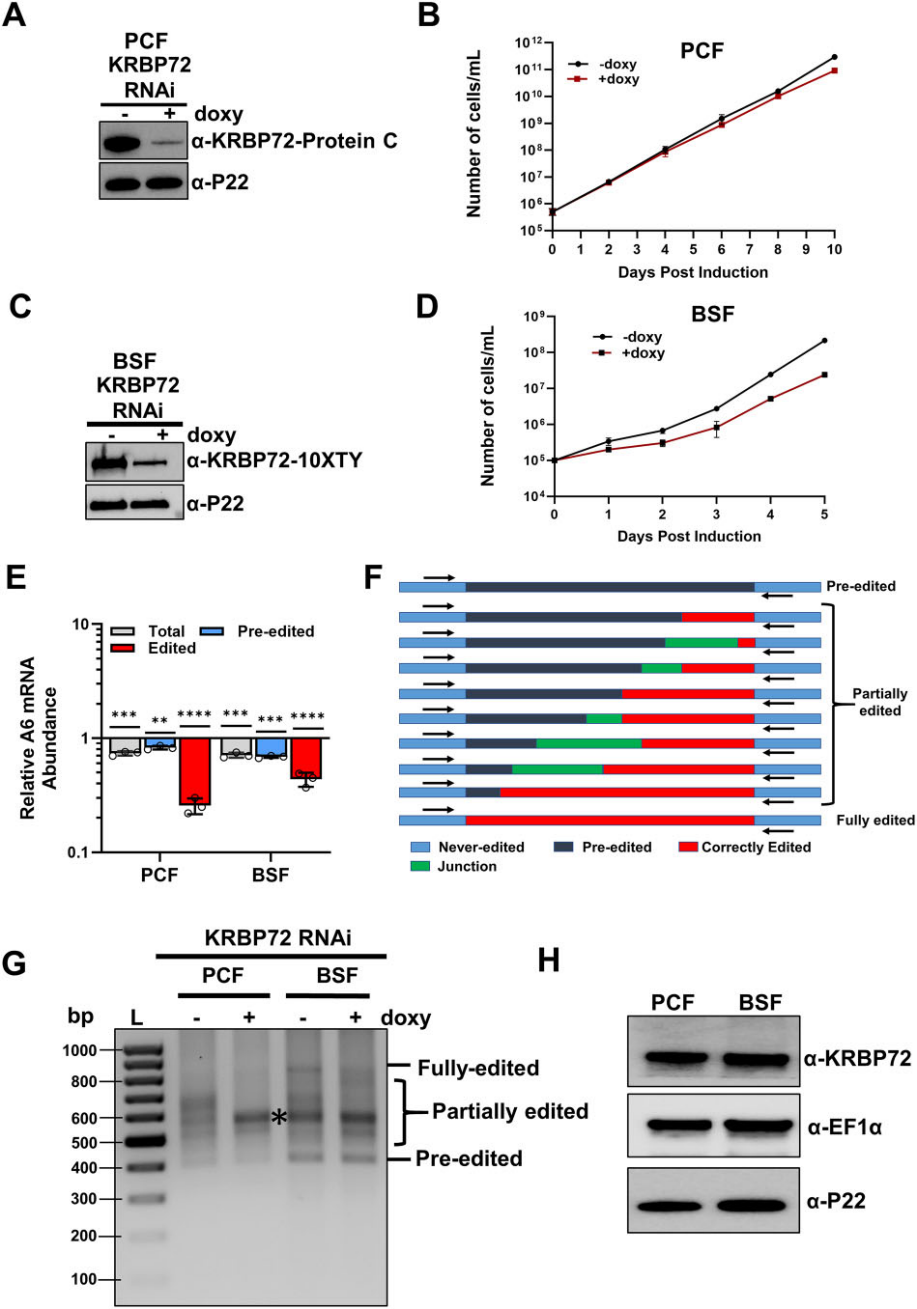
Effect of KRBP72 depletion and relative abundance of KRBP72 in two life cycle stages. (**A**) One allele of KRBP72 was PTP tagged in PCF *T. brucei*; western blot showing the degree of KRBP72 knockdown by immunoblot against the PTP tag. (**B**) KRBP72 was repressed using doxy-inducible RNAi in PCF *T. brucei*, and cell growth was monitored for 10 days in triplicate for both uninduced (−doxy) and induced cells (+doxy). We note that the uninduced cells grew somewhat more slowly than is typical for PCF cells. (**C**) One allele of KRBP72 was 10XTY tagged in BSF *T. brucei*; western blot showing the degree of KRBP72 knockdown by immunoblot against the TY tag. (**D**) KRBP72 was repressed using doxy-inducible RNAi in BSF *T. brucei*, and growth was monitored for 5 days in triplicate for uninduced (−doxy) and induced cells (+doxy). (**E**) RNA was isolated from both PCF and BSF forms of *T. brucei* on day 3 and day 2 post-induction, respectively. A6 mRNA levels were quantified by quantitative reverse transcription-polymerase chain reaction (qRT-PCR) using primer sets specific for total, pre-edited and fully edited RNAs. Relative RNA abundance represents levels in induced (+doxy) cells compared to levels in uninduced (−doxy) cells, normalized to 18S ribosomal RNA (rRNA) levels. Three biological replicates were performed, each with three technical replicates. Statistical significance of effects in induced cells compared to uninduced controls was evaluated was evaluated using Student’s *t*-test. **<0.01; ***P < 0.001; ****P < 0.0001. (**F**) Schematic representation of the full gene polymerase chain reaction (PCR) primers used to detect the largest pool of A6 mRNAs including pre-edited, fully edited and heterogenous partially edited RNAs. (**G**) Agarose gel analysis of A6 reverse transcriptase-polymerase chain reactions (RT-PCRs) using RNAs isolated from KRBP72-PTP/KRBP72 PCF RNAi cells and KRBP72-TY/KRBP72 BSF RNAi cells grown in the absence or presence of doxy for 3 days (PCF) or 2 days (BSF). Reverse transcription was carried out with an oligo(dT) primer, and PCR was performed with primers specific to the 5′ and 3′ ends of the A6 mRNA to amplify the entire population of mRNAs, including pre-edited, partially edited and fully edited. L, size ladder. (**H**) Western blot showing the relative abundance of KRBP72 in PCF and BSF cells. EF1α and P22 are controls.

**Figure 2.**
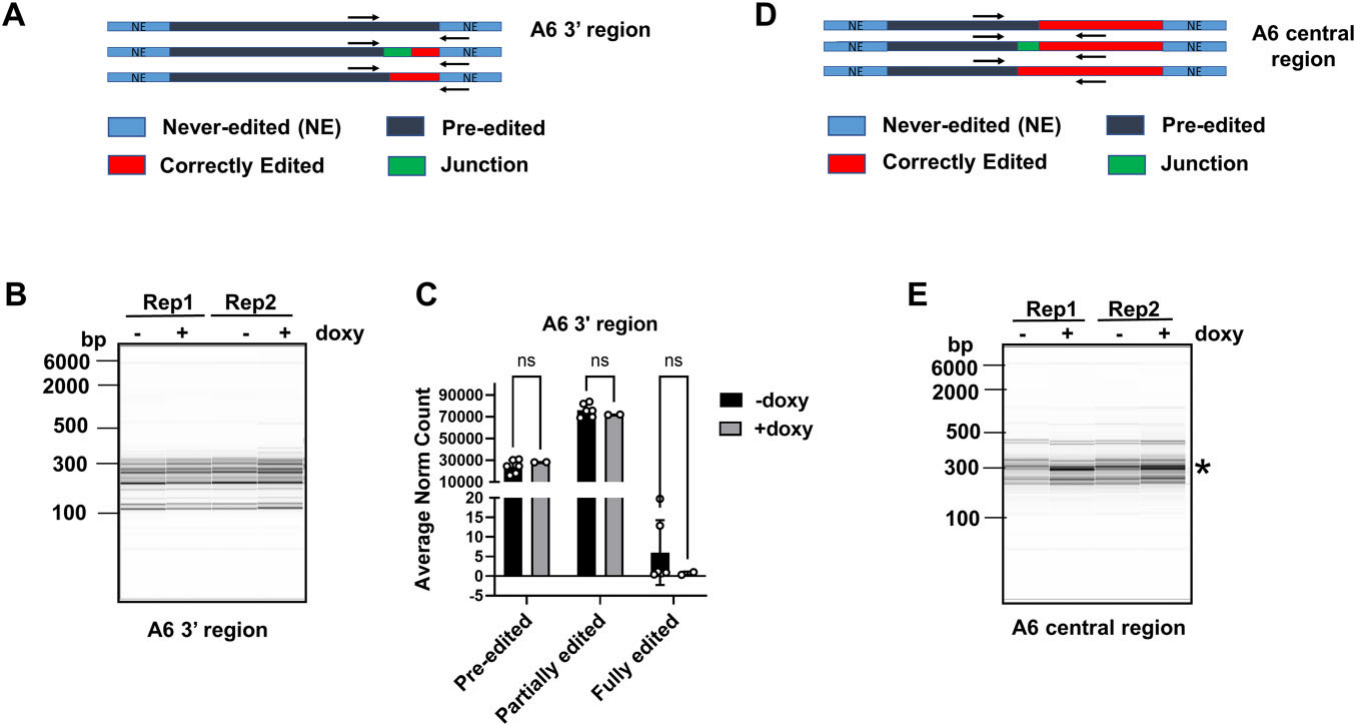
Capillary gel electrophoresis and high-throughputuence analysis of A6 mRNA in uninduced and induced PCF KRBP72 RNAi cells. (**A**) Schematic representation of the 3′ region primers used to detect the largest pool of A6 mRNAs in this region including pre-edited, fully edited and heterogenous partially edited RNAs. (**B**) Capillary gel electrophoresis of two biological replicates of uninduced (−doxy) and induced (+doxy) cells in the 3′ region. (**C**) Read counts for each sample were normalized to 100 000, and the average levels of normalized pre-edited, partially edited and fully edited A6 reads were calculated for uninduced (−doxy) and induced (+doxy) cell samples. Partially edited sequences are defined as reads that are not fully edited but have some U insertion or deletion; fully edited sequences are defined as reads with canonical fully edited sequence up to the 5′ primer used for amplification of the A6 mRNA 3′ end. ns, non-significant using Student’s *t*-test. (**D**) Schematic representation of the central region primers used to detect the largest pool of A6 mRNAs in this region, including pre-edited, fully edited and heterogenous partially edited RNAs. (**E**) Capillary gel electrophoresis of two biological replicates of uninduced (−doxy) and induced (+doxy) cells in the central region. Asterisk indicates a band representing an editing pause in induced KRBP72 RNAi cells that is absent in uninduced cells.

**Figure 3.**
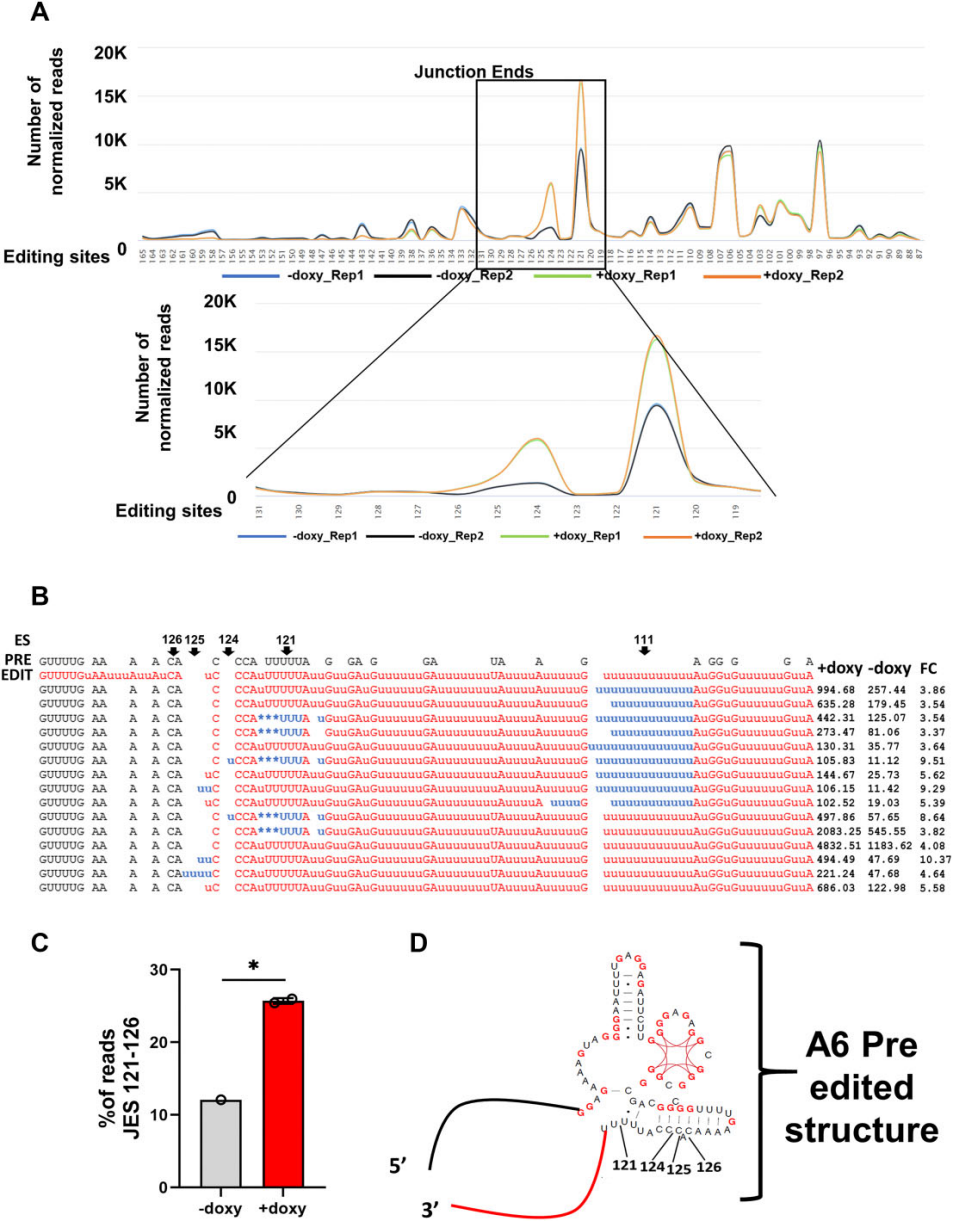
Editing profiles of A6 mRNA intermediates in uninduced and induced KRBP72 RNAi cells. (**A**) Junction End Site (JES) profiles for uninduced (−doxy) and induced (+doxy) cells for A6 mRNA intermediates. The X axes represent editing sites (ES). The Y axes represent the number of normalized read counts for mRNAs having a JES at a given ES. Top, ES 87–165; Bottom, expanded view of ES 119–131. Blue and black lines represent the two uninduced (−doxy) replicates; green and orange lines represent the two in induced (+doxy) replicates. (**B**) Sequences having the following characteristics are shown: a JES between ES121-126, at least a 3-fold difference between KRBP72 replete and depleted cells, and a minimum of 100 reads in the KRBP72 knockdown. These sequences are aligned with pre-edited (PRE) and fully edited (EDIT) A6 sequences. Editing Site; ES. Averaged normalized counts for induced (KRBP72) and uninduced (AvgUn) KRBP72 RNAi samples and fold change in induced relative to uninduced (fold change; FC) are shown to the right of each sequence. Black text, pre-edited sequence; red text, fully edited sequence; blue text, partially edited sequence. (**C**) Graph representing the percentage of reads at JES 121–126 in −doxy- and +doxy-treated cells. Statistical significance was determined using Student’s *t*-test, with * indicating *P* < 0.05. (**D**) Portion of A6 pre-edited mRNA structure showing locations of prominent JES. Structure adapted from (54) and published under a Creative Commons License (https://creativecommons.org/licenses/by/4.0/). G residues are in red to highlight the G quadruplex and G-C basepairs at base of 3′-most stem loop. Red line represents fully edited sequence and black line represents pre-edited sequence similar to sequences shown in Figure 3B.

*T. brucei* strain 29–13 and derivatives thereof were used for all PCF experiments. Cells were grown in at 27°C in SDM-79 medium supplemented with 10% fetal bovine serum (FBS). Cells harboring KRBP72-ProteinA-Tev-ProteinC (PTP) were previously reported (23). To generate a KRBP72-PTP/KRBP72 RNAi cell lines, the forward primer *KRBP72*_5′-F and reverse primer *KRBP72*_3′-R containing Bam*HI* and Hind*III* sites, respectively, were used to amplify a 1021 bp amplicon, which was ligated into pJET1.2/Blunt (Thermo Scientific) plasmids (Supplementary Table S1). The resulting plasmids were digested with Bam*HI* and Hind*III* and the resulting ∼1 kb fragment was ligated into p2T7-177 (37) between opposing tetracycline/doxycycline (doxy)-regulated T7 RNA polymerase promoters to give KRBP72_p2T7-177. The plasmid was NotI digested, purified and transfected into KRBP72-PTP cells. Cells were selected with phleomycin (2.5 μg/ml) and puromycin (1 μg/ml) and clones obtained by limited dilution. KRBP72-PTP/KRBP72 RNAi cell line was used for the experiments in Figures 1–3.

Monomorphic Lister 427 BSF single-marker parasites were cultured at 37°C with 5% CO_2_ in modified HMI-9 medium supplemented with 10% heat-inactivated FBS, 2.5 μg/ml G418 and 2.5 μg/ml hygromycin B. For KRBP72 RNAi BSF cell lines generation in this study, we used the p2T7-177 construct described above. Approximately 4 × 10^7^ BSF parasites were used for transfection. Transfected parasites were allowed to rest for 12–16 h. at 37°C with 5% CO_2_. The transfectant culture was then diluted 1:2, 1:5, 1:10 and 1:100 with fresh modified HMI-9 medium containing phleomycin (2.5 μg/ml) and aliquoted into 24-well plates. KRBP72 RNAi cell lines were maintained in phleomycin. To generate BSF KRBP72-10XTy and KRBP72 RNAi cell lines, the KRBP72 RNAi cell line described above was transfected with a 10XTy-tagging cassette obtained by amplification of the pPOTv4-MHT(Myc-His-TAP)-blasticidin vector with PCR primers (KRBP72_SD-F and KRBP72_SD-R) as described earlier (31,38). Cells were selected with phleomycin (2.5 μg/ml) and blasticidin (20 μg/ml), and clones obtained by limiting dilution.

To check the levels of KRBP72 depletion in RNAi lines, whole cell lysates of PCF and BSF trypanosomes were immunoblotted with anti-ProteinC antibody for PCF cells to detect the level of KRBP72-PTP or anti-Ty antibody for BSF cells to detect the level of KRBP72-10XTy; P22 (25) served as a control. Growth rates were analyzed for three biological replicates of each cell line. If error bars are not visible in growth curves, it is due to their small size compared to the symbol. To determine the abundance of KRBP72 in PCF versus BSF, equal number of whole cell lysates (5 × 10^5^) from PCF and BSF trypanosomes were loaded onto an sodium dodecyl sulphate-polyacrylamide gel electrophoresis (SDS-PAGE) gel, and a western blot was performed. Anti-KRBP72 antibody (see below) was used to detect the level of KRBP72, while EIF1α and P22 served as loading controls.

To generate a cell line for enhanced cross-linking and affinity purification (eCLAP), we established a KRBP72-MHT cell line in the 29–13 background. Cells were transfected with an MHT-tagging cassette obtained by amplification of the pPOTv4-MHT-Puromycin vector with PCR primers (KRBP72_SD-F and KRBP72 _SD-R). Transfectants were selected with puromycin (1 μg/ml) and clones obtained by limited dilution.

To generate the KRBP72 WT, RR-AA and K-A overexpression cell lines, we first used the online tool RecodeTryps (https://www.acsu.buffalo.edu/∼lread/tools.html) to recode *T. brucei* KRBP72 protein-coding sequence for optimal codon usage. All the constructs were synthesized by GenScript and contained an in-frame C-terminal 5X-TY tag. These constructs were ligated into pLEW100, resulting in integration into the rDNA spacer region (39). The resulting pLEW100_KRBP72(WT), pLEW100_KRBP72(RR-AA) and pLEW100_KRBP72(K-A) plasmids were *Not*I digested, purified and transfected into PCF *T. brucei* 29–13 cells. To generate cells with KRBP72 RNAi and complemented with the KRBP72 wild-type or mutant proteins, we first generated a PCF KRBP72 RNAi cells in the 29–13 background line generated using the KRBP72_p2T7-177 construct described above. The pLEW100_KRBP72 (WT), pLEW100_KRBP72(RR-AA) and pLEW100_KRBP72(K-A) plasmids described above were *Not*I digested, purified and transfected into the KRBP72 RNAi cell line in the 29–13 background. For all cells harboring pLEW100-based plasmids, cells were selected with puromycin (1 μg/ml) and clones obtained by limiting dilution. Growth rates were analyzed for three biological replicates of each cell line.

To generate a cell line with both RESC12A-MHT and KRBP72 RNAi, the PCF KRBP72 RNAi cell line in the 29–13 background was transfected with a MHT-tagging cassette obtained by amplification of the pPOTv4-MHT-Puromycin vector with PCR primers (RESC12_SD-F and RESC12_SD-R) as described (31,38). To generate the KRBP72-10XTY cell line used for co-immunoprecipitations, PCF strain 29–13 was transfected with a 10XTY-tagging cassette obtained by amplification of the pPOTv4-MHT-blasticidin vector with PCR primers (KRBP72_SD-F and KRBP72_SD-R). The RESC12/12A RNAi and RESC6 RNAi lines are previously published (34,40).

### Full gene RT-PCR analysis of A6 transcripts

PCF KRBP72-PTP/KRBP72 RNAi cell lines were grown in the presence or absence of 4 μg/ml doxy for 3 days and BSF *T. brucei* KRBP72-10X-Ty tagged KRBP72 RNAi cell lines were grown in the presence or absence of 4 μg/ml doxy for 2 days. Complementing cell lines were also grown for 3 days in presence and absence of doxy. RNA was extracted and full gene RT-PCR analysis was performed as described earlier (23).

### Anti-KRBP72 antibody generation

The amplification of the KRBP72 gene from genomic DNA involved the use of pET28a_KRBP72-F and pET28a_KRBP72-R primers (Supplementary Table S1**primers**), incorporating *Bam*HI and *Eco*RI restriction overhangs in their 5′ ends, respectively. The amplified gene was then cloned into the pET28a expression vector (Novagen). Following sequence confirmation, the resulting plasmid was introduced into BL21 *Escherichia coli*. The recombinant KRBP72-6His protein was purified using Ni-NTA resin (GE Healthcare) according to the manufacturer’s recommendations and subsequently dialyzed in 1× phosphate-buffered saline (PBS) overnight.

Antibodies against KRBP72 were generated in rabbits at Bethyl Laboratories (Montgomery, TX, USA). For the affinity purification of anti-KRBP72 antibodies, KRBP72-6His (500 μg) was mixed with 2X sodium dodecyl sulphate (SDS) loading dye, transferred to nitrocellulose and the membrane was stained with Ponceau (Sigma). The KRBP72-6His band was excised and incubated overnight with α-KRBP72-containing rabbit serum at 4°C. After removal of the serum, the membrane was washed with 1X PBS with Tween-20 (PBST), and antibodies were eluted with 0.1 mM glycine (pH 2.5), followed by neutralization with 1 mM Tris-HCl (pH 8.0). The specificity of the antibody was confirmed by probing an RNAi cell line targeting KRBP72 and harboring one KRBP72-PTP allele as shown in Supplementary Figure S1.

### qRT-PCR analysis

KRBP72 RNAi cells were exposed to 4 μg/ml doxy for a 3-day period to induce RNAi, while a control group remained untreated. An equal number of cells were harvested from both conditions, and RNA extraction was carried out using Trizol (Ambion). Subsequently, the RNA underwent DNase treatment (Ambion) for 1 h, followed by purification through phenol/chloroform extraction and ethanol precipitation. RNA purity was assessed using a Nanodrop 1000, with a 260/280 ratio of approximately 2. RNA quality was verified on a 1.0% TBE (Tris/Borate/EDTA) agarose gel. Complementary DNA (cDNA) synthesis was performed using random hexamer primers and the iScript reverse transcriptase (RT) kit (Bio-Rad). Western blot analysis, employing PTP or TY antibody, or a KRBP72-specific antibody, was conducted to determine the levels of KRBP72 protein. qRT-PCR was carried out using established primers (14,15,41) to quantify total, pre-edited and edited mitochondrial transcripts from *T. brucei*. The data were normalized to 18S rRNA (41,42). Analysis of qRT-PCR results was performed using Bio-Rad iQ5 software. All the results reflect three biological replicates, except for Figure 5C, in which four biological replicates were performed, each with three technical replicates of the qRT-PCR reaction.

### High-throughput sequence analysis

PCF *T. brucei* KRBP72-PTP*/*KRBP72 RNAi cell lines were grown in the presence or absence of 4 μg/ml doxy for 3 days. RNA was isolated using Trizol followed by phenol/chloroform extraction and ethanol precipitation. RNA samples were treated with DNase and phenol/chloroform extracted and ethanol precipitated. Two biological replicate experiments were performed, and western blot was used to validate the level of KRBP72 knockdown. cDNA was generated from DNase-treated RNA using gene-specific primers (4). These cDNA samples were PCR amplified within the linear range of PCR to maintain the relative abundance of unique fragments. PCR amplicons were resolved on the Agilent 5200 Fragment Analyzer using the Agilent High Sensitivity NGS gel assay prior to sequencing. Paired-end Illumina Mi-Seq was used for high-throughput sequencing of amplicons as described previously (4). All reads were aligned to the published pre-edited and fully edited mRNA sequences (43). Reads with non-T SNPs or insertions/deletions relative to the published canonical sequence were excluded from the analysis (Supplementary Table S2). All remaining reads in each sample were normalized to 100 000 counts in order to compare relative abundances of specific sequences between samples. Exacerbated Junction End Sites (EJES) were calculated as described (5) (Supplementary Table S3). The sequencing data used in this study have been deposited in the Sequence Read Archive, PRJNA1133815.

### Immunoprecipitation

RESC12A-MHT tagged PCF cells harboring KRBP72 RNAi constructs were grown for 3 days following induction with 4 μg/ml doxy. Cells (1 × 10^10^) were collected and washed with 1× PBS. The cell pellet was lysed in N150 buffer [50 mM Tris-Cl (pH 7.5), 150 mM NaCl, 0.1% NP-40, 1 mM MgCl_2_ and 5 mM β-ME)] with 1% (*v/v*) Triton X-100 and 1 μg/ml DNase 1 by incubation for 30 min. The cell suspension was centrifuged at 18 000 rpm for 30 min. Supernatants from RESC12A-MHT were incubated in 20 ml Bio-Rad columns with 200 μl of IgG beads (GE Healthcare) for 3 h at 4°C. The beads were washed with 10 ml N150 buffer and followed by 10 ml tobacco etch virus (TEV) cleavage buffer [10 mM Tris-Cl (pH 7.5),150 mM NaCl, 0.5 mM ethylenediaminetetraacetic acid (EDTA)] and incubated in 1ml of TEV cleavage buffer with 10 μl of TEV protease (Thermo Scientific) overnight at 4°C. KRBP72 immunoprecipitation was carried out employing a similar approach, utilizing 2 ×10^10^ PCF *T. brucei* 29–13 cells. The cell lysate was incubated with Protein A fast flow beads (GE Healthcare) pre-bound to α-TY antibodies. Before incubation, the lysate was divided into two 20 ml fractions. One fraction was treated with 60 U of RNase inhibitor (Applied Biosystems) and DNase 1 (1 μg/ml), while the other fraction, in addition to DNase1, was subjected to a nuclease cocktail comprising 8 μg RNase A (Thermo Scientific), 2500 U RNase T1 (Ambion), 28 U RNase H (Invitrogen) and 2040 U micrococcal nuclease (Thermo Scientific) for 1 h on ice. Following washing with N150 buffer, KRBP72 containing complexes were eluted with 0.1 mM glycine (pH 2.5) and neutralized with 1 mM Tris-HCl (pH 8.0). Target protein levels were normalized by western blot analysis prior to probing with specific antibodies against the respective proteins: RESC6 (35), RESC13 (42), RESC11A (5), RESC12A (34), RESC8 (15), RESC14 (14), RESC10 (13), P22 (25) and RESC2 (40).

### Enhanced cross-linking and affinity purification

#### Cell collection and sample preparation

KRBP72-MHT tagged PCF cells were grown to ∼1 × 10^7^ cells/ml, and 3 × 10^9^ cells were collected per experiment. Cells were pelleted at 5000 *g* for 10 min, washed with ice-cold PBS containing 6 mM sucrose, and resuspended in 32 ml of the same buffer and centrifuged again. Cells were resuspended in in 50 ml SM media without FBS, distributed into two prechilled 10 cm Petri dishes, and crosslinked by irradiation at 800 mJ/cm^2^ twice using a Stratalinker Crosslinker. Crosslinked cells were centrifuged at 6000 *g* for 10 min at 4°C, resuspended in DTE buffer (1 mM Tris (pH 8), 1 mM EDTA, complete protease inhibitor cocktail, RNaseOUT), homogenized on ice and mixed with 60% sucrose before centrifuging at 15 800 *g* for 10 min. The pellet was resuspended in 7.5 ml STM buffer [250 mM sucrose, 20 mM Tris (pH 8), 2 mM MgCl_2_, 0.2 mM CaCl_2_] with DNase I and incubated on ice for 1 h, followed by the addition of 7.5 ml STE buffer [250 mM sucrose, 20 mM Tris (pH 8), 10 mM EDTA] and centrifugation at 13 000 rpm. The pellet was flash-frozen in liquid nitrogen and stored at −80°C. For lysis, the pellet was resuspended in lysis buffer [50 mM Tris (pH 8), 150 mM NaCl, 0.5% NP-40, 1% Triton X-100, Complete protease inhibitor cocktail, RNase Inhibitor], rotated at room temperature for 20 min, centrifuged at 13 000 rpm and the supernatant was collected. For RNase treatment, reactions in 1 ml volume included DNase TURBO, 1M CaCl2 and RNase I at specified dilutions, incubated at 37°C in a thermomixer at 1500 rpm for 5 min, followed by termination with EDTA and RNase inhibitor. For immunoprecipitation, either nickel-NTA agarose beads (Invitrogen) or HisPur™ Ni-NTA Magnetic (Thermofisher Scientific) were equilibrated in binding buffer, added to the lysate, rocked at 4°C for 2 h, washed with N150 buffer and incubated with denaturation buffer (50 mM Tris (pH 8), 150 mM NaCl, 0.5% NP-40, 8 M urea with complete protease inhibitor cocktail). Beads were washed with lysis buffer, then resuspended in Calf Intestinal Alkaline Phosphatase (CIAP) buffer with CIAP for 20 min at 37°C, washed with polynucleotide kinase (PNK) buffer and resuspended in T4 PNK buffer with ^32^P-[γ-ATP] and T4 PNK for 30 min at 37°C. After adding 1 mM ATP and incubating, beads were washed, resuspended in SDS loading buffer, boiled and the supernatant was loaded onto an SDS-PAGE gel for Western blotting, with the membrane exposed to a phosphor screen before antibody blotting against the myc tag.

#### Sequencing alignment to finished peak matrix

eCLAP libraries were prepared according to the RBP-eCLIP kit v1.01R (Eclipse Bioinnovations) protocol with modifications. Library quality was assessed using an Agilent Bioanalyzer, followed by single-end sequencing on an Illumina NextSeq High Output Single End 75 cycles at the CWRU Genomics Core. Specifically, all samples were crosslinked on ice with ultraviolet light in a Stratalinker. Cells were then harvested and lysed by sonication. The lysates were then treated with DNase and RNase followed by affinity purification of the MHT-tagged protein via anti-HIS Dynabeads. After washing the bead bound samples, the 3′ end adapter was ligated to the RNA and then the RNA-protein complex was purified by SDS-PAGE and transferred to nitrocellulose membrane. RNA liberated from the nitrocellulose was subject to first strand synthesis, ligation of second adapter and amplified by PCR. Size-matched input processing and final cDNA library gel purification steps were omitted while adding an additional round of AMPure PCR cleanup to purify the final libraries.

FASTQ files were processed using CLIPittyClip pipeline as described before (44). Briefly, demultiplexed FASTQ files were sequentially processed using fastx_toolkit (https://www.hannonlab.org/resources/) and CLIP Tool Kit (CTK) (45). The reads were first quality filtered and identical reads were collapsed to reduce redundancy and increase computational efficiency. Then, barcode and adapter sequences were trimmed from 5′ and 3′ end of the reads. Processed reads at least 16nt long were kept for alignment. Processed reads were mapped to a curated *T. brucei* mitochondrial genome containing published fully edited, pre-edited and never edited sequences as well as to a nuclear encoded gene file comprising the TriTrypv46 gene annotation file modified to extend each transcript at its 3′ end to the annotated 5′ end of the downstream gene (46) using Bowtie2 (47) allowing up to one mismatch. The alignment results in SAM format were converted to BAM format using Samtools (48), which were then used as inputs for BEDTools (49) to generate mapped read files in BED format and mapped read coverage files in BEDGraph format.

#### Tentative crosslink site analysis

Using mapped reads, tentative crosslink sites were identified as 1 nucleotide upstream of the 5′-end of the reads. Peak calling was performed using HOMER (50) using a compiled BED file containing all crosslink sites. Using HOMER, a tag directory was first generated, and peak calling was performed with the following parameters: -style factor -L 2 -local Size 10 000 -strand separate -minDist 20 -size 15 -fragLength 25. Lastly, a stringent filter based on the biological complexity (i.e. replicate counts) and read densities were applied to the peak output file to exclude any peaks that were not present in at least two replicates.

### RNA immunoprecipitation

PCF cells expressing KRBP72 WT, RR-AA or K-A in the KRBP72 RNAi background (see above) were grown in the presence of 4 μg/ml of doxy for 3 days. Cells (1 × 10^10^) were collected, mitochondria were enriched and RNA immunoprecipitation (RIP) was performed as described previously using α-TY antibody crosslinked to Protein A beads (13,14,51,52). RIP of RESC12/12A and RESC6 cells grown in the absence or presence of 4 μg/ml of doxy for 3 days was performed similarly using α-KRBP72 antibody crosslinked to Protein A beads. Five percent of the beads was taken from each sample, and a western blot was performed to confirm the pulldown of KRBP72 proteins. The supernatant was removed after DNase1 (Sigma) treatment followed by proteinase K (Roche) treatment. RNA was extracted with phenol/chloroform followed by ethanol precipitation. RNA was DNase-treated and 500 ng of RNA converted to cDNA with established gene specific primers targeting total mRNA A6 and 18S rRNA (14,53) using the iScript cDNA synthesis kit (Bio-Rad). cDNA was amplified using SsoAdvanced PreAmp Supermix (Bio-Rad), and then used for qRT-PCR, with 18S rRNA used for normalization. The ΔΔCt method was used to determine the fold change as described previously (14).

### Vienna RNA structure prediction

Partially edited A6 mRNA structure prediction was performed with Vienna RNA version 2.4. The A6 mRNA sequence, fully edited to ES 124 and pre-edited 5′ of that point, was analyzed with the RNAfold program under a temperature setting of 27°C.

## Results

### Life cycle specific effects of KRBP72 on A6 mRNA editing

We previously reported that KRBP72 depletion affects the editing of A6 mRNA in PCF cells due to pausing at a specific region, while other transcripts remained essentially unaffected (23). Our present study aims to investigate the specific mechanism by which KRBP72 affects U-indel editing. To begin to address this question, we generated a new PCF KRBP72 RNAi cell line in which one allele of KRBP72 is PTP tagged, and confirmed >95% knockdown through western blot analysis using a Protein C antibody (Figure 1A). We note that this level of KRBP72 depletion exceeds that of the previous study in which 50% depletion at the mRNA level was achieved. Nevertheless, we observed little effect of KRBP72 depletion on PCF cell growth (Figure 1B). To determine if KRBP72 function is conserved during the *T. brucei* life cycle, we established a similar RNAi cell line harboring endogenously TY-tagged KRBP72 in BSF (Figure 1C). Again, only a modest effect of KRBP72 RNAi on cell growth was observed, albeit slightly more pronounced than that in PCF (Figure 1D). We next analyzed the effect of KRBP72 depletion on mRNA editing and abundance in both life cycle stages using established primer sets (14,15,41,53). With regard to A6 mRNA, both PCF and BSF exhibited significantly decreased edited mRNA upon KRBP72 RNAi induction, with edited A6 mRNA levels in PCF at ∼25% and BSF at ∼50% of edited A6 mRNA levels in uninduced cells (Figure 1E). The corresponding total and pre-edited mRNAs were also very modestly decreased (Figure 1E). While the small decrease in total mRNA levels suggests an effect of KRBP72 on A6 mRNA stability, the substantially larger decrease in edited A6 mRNA levels suggests that KRBP72 also promotes A6 mRNA editing in both life cycle stages (Figure 1E). In BSF, no other effects were observed (Supplementary Figure S2). In PCF, the levels of both total and edited RPS12, CYb and COIII mRNAs were modestly decreased (Supplementary Figure S2). Because total mRNAs were decreased to similar levels as edited mRNAs, this result is more consistent with KRBP72 having an impact on the stability of these RNAs that secondarily leads to decreased edited mRNA levels. Together, these data demonstrate that the effects of KRBP72 on U-indel editing are restricted to A6 mRNA, and these effects are manifest in both PCF and BSF life cycle stages.

We next asked if KRBP72 is involved in progression through the same pause site in A6 mRNA in PCF and BSF. To this end, we conducted a full gene PCR assay, utilizing primers corresponding to the never-edited regions of A6 transcripts (Figure 1F) (23,26). Because U insertion is much more frequent than U deletion, increased editing can be visualized by electrophoresis as larger products in the full gene PCR. In PCF cells, depletion of KRBP72 resulted in the accumulation of a prominent band just under 600 bp, indicating an accumulation of distinct partially edited transcripts and an effect on editing progression as previously observed (Figure 1G) (23). The absence of pre-edited mRNA accumulation confirms that A6 mRNA editing initiation remains unaffected. When we compared the PCF and BSF RNAi cell lines, we found that the most prominent band in uninduced BSF cells corresponded to that induced by KRBP72 knockdown in PCF, and the intensity of this band was not substantially affected by KRBP72 knockdown in BSF. Rather, we noted a decrease in fully edited transcripts and modest accumulation of other partially edited products in induced BSF cells (Figure 1G). These differences led us to hypothesize that KRBP72 abundance might differ between these cell lines. To investigate this, we generated an antibody against KRBP72 and performed western blots, which showed that the levels of KRBP72 were consistent between PCF and BSF cells (Figure 1H and Supplementary Figure S1). Therefore, we conclude that KRBP72 levels do not play a role in the observed life cycle specific differences in A6 mRNA patterns. Overall, our findings indicate the presence of two distinct patterns of A6 mRNA editing between PCF and BSF cells, with the knockdown of KRBP72 in PCF cells yielding more pronounced effects on A6 editing compared to BSF cells. As a result, we decided to focus our subsequent investigations on the PCF life cycle stage.

### KRBP72 promotes the progression of A6 RNA editing past a predicted stem loop element

To confirm that KRBP72 affects editing progression, but not initiation, and to identify the region within A6 mRNA that requires this factor for its transversal, we employed Illumina MiSeq sequencing and applied the Trypanosome RNA Editing Alignment Tool (TREAT) for analysis. TREAT enables us to quantify the ratios of pre-edited, partially edited, and fully edited mRNAs while also allowing for the examination of single nucleotide level details within partially edited mRNA sequences (5). Due to the impracticality of sequencing the entire edited domain of the A6 mRNA using MiSeq, we begin by amplifying, within the linear range, its 3′ edited region using a forward primer designed to target the pre-edited sequence near the middle of the transcript and a reverse primer located in the 3′ never-edited region (Figure 2A, Supplementary Figure S3 (green) and Supplementary Table S1) (53). In the context of this primer placement, the term "fully edited’ mRNA refers to mRNA intermediates that have been fully edited up to the pre-edited forward primer. High resolution fragment analysis of 3′ region PCR products revealed no differences between uninduced and induced samples (Figure 2B), indicating that neither A6 mRNA editing initiation nor progression of editing within the 3′ region of the transcript is affected by KRBP72 depletion. TREAT analysis of 3′ region reads confirmed that the levels of pre-edited transcripts, and thus editing initiation, were unaffected by KRBP72 knockdown (Figure 2C).

The above results indicated that the region of pausing in KRBP72 knockdown cells lies 5′ of ES 114 (Supplementary Figure S3). Based on these results, we a designed a new primer set for amplification of the A6 mRNA central region (Figure 2D, Supplementary Figure S3 (purple) and Supplementary Table S1). Fragment analysis of central region amplicons identified a prominent product of approximately 300 base pairs in KRBP72 depleted cells (Figure 2E), indicating that the pause site lies between the regions covered by this primer pair. We, thus, subjected these amplicons to high-throughput TREAT analysis to define the pause region at single nucleotide resolution. As depicted in Figure 2A and D, the 5′ most boundary of editing may be either the 5′ most correctly edited site or it may be the 5′ end of a junction region (Figure 2A and D, green). Junctions are frequently observed at the boundaries of correctly edited and pre-edited sequences, and many junctions are thought to represent regions of active editing (3). To analyze the exact location at which the editing process terminates upon KRBP72 depletion, we identified EJES in duplicate KRBP72 RNAi cells, comparing these to the duplicate uninduced cells, as previously described (5). The TREAT platform defines the Junction Start Site as the initial ES that, moving 3′ to 5′, fails to correctly match the canonical fully edited sequence (it can match pre-edited or non-canonically edited sequences) (4). The JES is characterized as the 5′ most ES with any editing action, whether canonical or non-canonical. Using this approach, we identified 10 EJES in the KRBP72 knockdowns compared to their uninduced counterparts (Figure 3A, Supplementary Figure S4 and Supplementary Table S3). Figure 3A shows the number of normalized reads at each ES across the A6 transcript in duplicate uninduced and doxy-induced KRBP72 RNAi cells. This EJES analysis revealed that a major region of pausing in the editing process occurs in KRBP72 depleted cells at a region spanning ES121-126, with sequences having a JES at ES121 and ES124 making up the bulk of these sequences (Figure 3A, Supplementary Figure S4 and Supplementary Table S3). From these analyses we conclude that a region between ES121-126 of A6 mRNA relies upon KRBP72 for efficient editing progression.

To further define the precise nature of the editing defect in KRBP72 depleted cells, we next analyzed our high-throughput data at the single nucleotide level. We identified those sequences having JES between ES121-126, with at least a 3-fold difference between KRBP72 replete and depleted cells and a minimum of 100 reads in the KRBP72 knockdown. Fifteen sequences met these criteria (Figure 3B). We noted that nine of these sequences contained a non-canonical sequence at ES111, for which the canonical sequence is 12 U insertions; however, in all but one case, these were correctly edited for five ES 5′ of this site. Thus, ES111 appears to present a substantial challenge to the editing machinery, beyond which editing can continue accurately until it is again interrupted in the KRBP72 knockdown between ES121-126. In addition, a large number of reads stall between ES121-126 after continuous correct editing throughout the transcript. In all, the percentage of cells with JES between ES121-126 increased from 12.1% in uninduced cells to 25.7% upon knockdown of KRBP72 (Figure 3C), with individual JES contributing differently to this increase (Supplementary Figure S4). Overall, these data confirm that the depletion of KRBP72 results in a roadblock in A6 mRNA editing between ES121-126, with many, but not all, transcripts containing a few sites of non-canonical editing within this region.

We next asked if the structural context of this region of the A6 transcript contributes to its need for an auxiliary factor for editing progression. Leeder *et al.* previously published structures of pre-edited mRNAs determined by selective 2′-hydroxyl acylation analyzed by primer extension (SHAPE) in combination with RT stop assays to identify G-quadruplexes (54,55). Because the A6 mRNA sequence 5′ of the identified pause sites is pre-edited, we reasoned that analysis of editing pauses in the context of the pre-edited A6 mRNA structure would be informative. Figure 3D shows the predicted pre-edited A6 mRNA structure in this region, including an experimentally verified G-quadruplex, near the region of pausing. Moving in the 3′ to 5′ direction of editing, the G-quadruplex is preceded by a stem loop structure, containing four contiguous G-C base pairs. ES121, which constitutes a major pause site in KRBP72 depleted cells, lies at the base of this stem. In many sequences with JES121, editing proceeds correctly through ES121 and then stops; in others, an alternative non-canonical editing pattern is observed at ES120/121, after which editing does not proceed (Figure 3B). For those sequences with JES124 or JES125, some U insertion proceeds within the stem, near the site of a bulged A, after which editing terminates. Overall, our high-throughput sequence data analyzed in the context of A6 mRNA structure suggests that KRBP72 is critical for editing progression through a series of G-C basepairs at the base of a stable stem loop structure in the pre-edited region of A6 mRNA.

### eCLAP reveals KRBP72 binding sites on A6 pre-edited mRNA

Having provided evidence that editing pauses at the base of the stem loop at ES 121–126, we next investigated where KRBP72 binds on A6 mRNA relative to the pause sites. To facilitate this, we endogenously tagged KRBP72 with a MHT tag, creating the KRBP72-MHT cell line. To identify high-confidence binding sites for KRBP72 across the transcriptome, we performed single-nucleotide resolution eCLAP. To this end, we excised the region of gel up to ∼100 nucleotides above the cross-linked KRBP72 band (Supplementary Figure S5) in triplicate samples, and subjected the eluted fragments to high-throughput sequencing. We obtained quantitative estimates of KRBP72 binding intensity using HOMER software, which revealed log2 fold intensity in all the replicates. This resulted in 40 and 61 025 high-confidence peaks in mitochondrial and nuclear encoded transcriptomes, respectively (Supplementary Tables S4 and S5). Despite the clear mitochondrial function and protein-protein interactions of KRBP72, binding to non-mitochondrial RNAs was not unexpected. TrypTag indicates KRBP72 localization in cytoplasm (patchy) and nucleoplasm (pointed) (56), strongly suggesting multiple localizations and functions for this protein. In this study, we focused our analysis on the mitochondrial transcriptome; analysis of non-mitochondrial eCLAP hits will be reported elsewhere.

**Figure 4.**
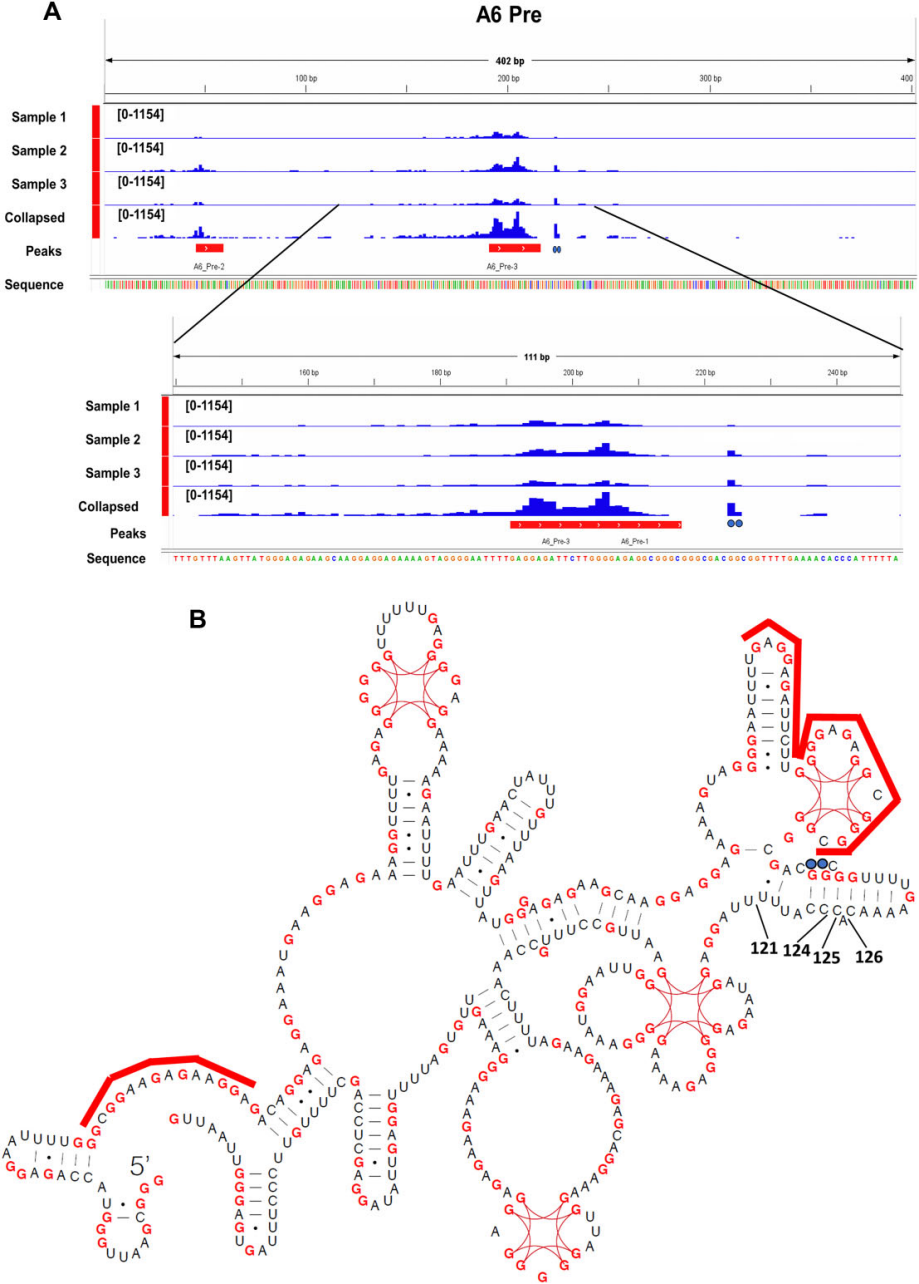
eCLAP analysis of KRBP72 on A6 mRNA. (**A**) KRBP72 binding sites are shown by blue bars. Two high confidence peaks are indicated by the red bars; two binding sites within the stem loop at which editing pauses in the KRBP72 knockdown are denoted by blue dots. Three biological replicates (Samples 1–3) and combined binding sites (Collapsed) are shown. Top, ES 1–306. Line colors indicate bases: A, green; red, T; blue,C; orange, G. Bottom, expanded view of ES 121–203 showing base identities with the same color scheme as the top panel. (**B**) A6 pre-edited mRNA structure adapted from (54) and published under a Creative Commons License (https://creativecommons.org/licenses/by/4.0/). Red lines above the structure denotes the two high confidence peaks; blue dots indicate binding sites within the stem loop at which editing pauses in the KRBP72 knockdown.

Mapping the location of KRBP72 binding sites across the mitochondrial transcriptome revealed no high confidence peaks in never edited or minimally edited mRNAs (Supplementary Figure S6). In pan-edited mRNAs, the majority of high confidence peaks (14/17) were in pre-edited sequences, with few peaks in the corresponding fully edited sequences (Supplementary Figure S6). We also found a large number of binding sites for 9S and 12S ribosomal RNA, which we hypothesize is due to the high abundance of these RNAs (Supplementary Figure S6). We were particularly interested in whether the sites of editing pausing in A6 mRNA in KRBP72 RNAi cells are related to KRBP72 binding. Again, no peaks were observed in fully edited A6 mRNA. However, two high confidence peaks were observed in pre-edited A6 mRNA. A prominent peak was found near the region at which we observed the pausing upon KRBP72 RNAi knockdown, and a smaller peak was found at the 5′ end of the transcript (Figure 4A and B, red lines). Alignment of pausing-proximal binding sites with the A6 pre-edited structure showed that the prominent high confidence peak aligns to G-quadruplex and stem loop structures 5′ of the pausing site. In addition, two binding sites represented by relatively high numbers of reads were observed in the predicted stem loop that causes pausing of editing in the KRBP72 RNAi cells (Figure 4A and B, blue dots). These data suggest that the modest direct KRBP72 binding on the stem loop that contains the pause sites ES121-126 permits editing progression through this structure. Alternatively, KRBP72 bound to the G-quadruplex is in a 3D position that allows it to transiently bind and impact editing in the ES121-126 containing stem loop. As in A6 pre-edited mRNA (Figure 4B), high confidence KRBP72 binding sites across other pre-edited mRNAs included numerous features, with selected G-quadruplexes, stem loops and single stranded regions demonstrating KRBP72 binding.

### Functional significance of KRBP72 RNA-binding and ATPase domains in A6 mRNA editing

KRBP72 harbors both a central ABC ATPase domain and an N-terminal region in which arginines 198 and 199 are critical for RNA binding (23). To better understand the mechanism by which KRBP72 impacts A6 mRNA editing, we investigated the contributions of both RNA-binding and ATPase activities to the progression of editing across the ES121-126 region. We began by overexpressing TY-tagged wild type (WT OE) and two mutant variants of KRBP72: an RNA-binding mutant with arginine to alanine substitutions at amino acids 198/199 (RR-AA OE) and an ATPase domain mutant with a lysine to alanine mutation at amino acid 267 (K-A OE) that is predicted to abolish ATPase activity (23,57,58) (Supplementary Figure S7A). We reasoned that overexpression of non-functional mutants might cause a dominant negative effect on A6 mRNA editing if either RNA-binding or ATPase activities were essential for this KRBP72 function. Cell lines in which wild-type or mutant KRBP72 was expressed to a similar extent as endogenous protein (i.e. 2-fold overexpressed) were generated (Supplementary Figure S7A). We then monitored A6 mRNA levels and cell proliferation (Supplementary Figure S7B and C). However, we observed no effects of overexpression on editing of A6 mRNAs (Supplementary Figure S7B), nor were there any observed growth defects (Supplementary Figure S7C). The absence of editing and growth defects upon KRBP72 mutant overexpression could be due to the homodimeric nature of KRBP72, in which inclusion of one mutant subunit may not substantially affect function.

**Figure 5.**
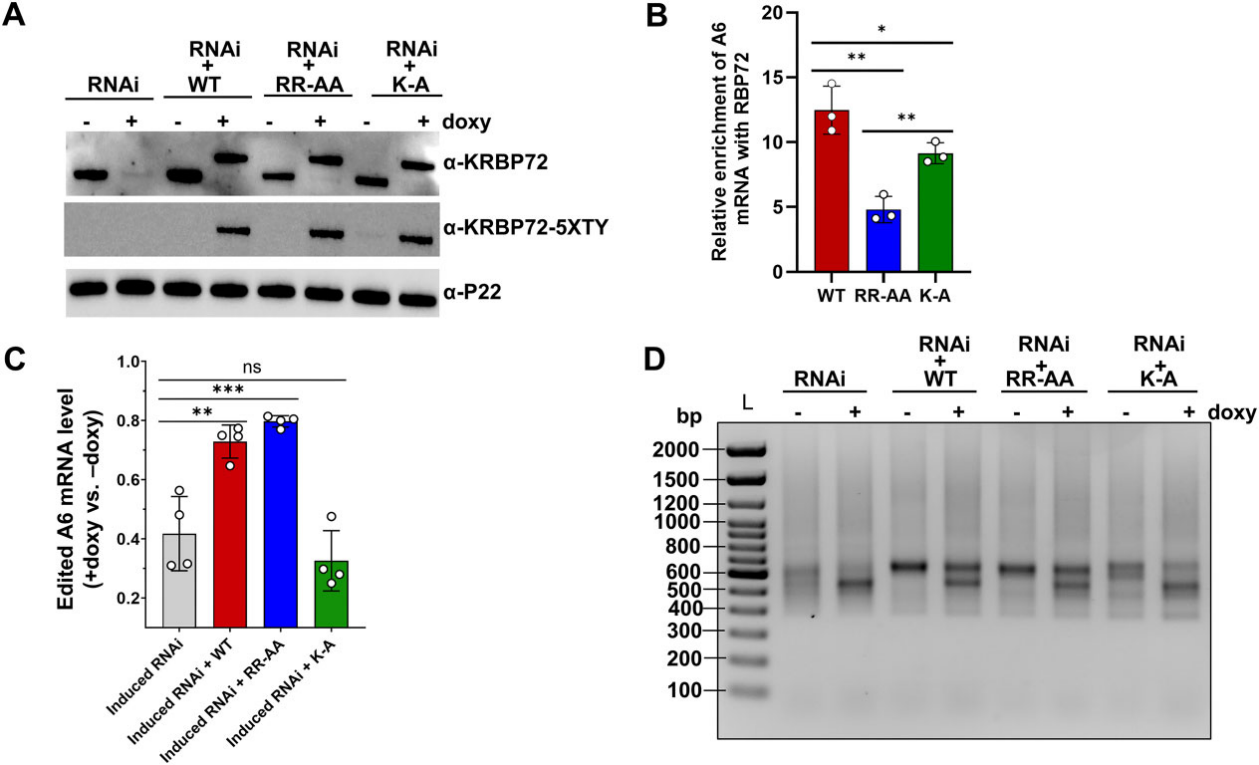
Complementation of KRBP72 RNAi cells with wild-type and mutant proteins. (**A**) Western blot with α-KRBP72 antibody showing the simultaneous doxy-induced KRBP72 RNAi and expression of TY-tagged WT or mutant KRBP72. Cells were collected 3 days post-induction. Expression of exogenous proteins was also confirmed using α-TY antibody. P22, loading control. (**B**) RIP showing enrichment of total A6 transcripts in KRBP72 WT, KRBP72 RR-AA and KRBP72 K-A cells by α-KRBP72 antibody-mediated immunoprecipitations relative to a negative antibody control. Immunoprecipited RNA was measured by qRT-PCR with 18S rRNA was used for normalization. Values represent the mean of three biological replicates, each with three technical replicates. Significance was evaluated using Student’s *t*-test. ***P < 0.001; ****P < 0.0001. (**C**) Edited A6 mRNA levels were quantitated by qRT-PCR. RNA was isolated from KRBP72 RNAi cells and all three complemented cell lines on day 3 post-induction. Relative RNA abundance represents levels in induced (+doxy) cells (KRBP72 RNAi or KRBP72 RNAi complemented with either WT, RR-AA, or K-A KRBP72) compared to levels in uninduced (−doxy) cells, normalized to 18S rRNA levels. Four biological replicates were performed, each with three technical replicates. Significance was evaluated using Student’s *t*-test. ****P < 0.0001. (**D**) Agarose gel analysis of A6 RT-PCR reactions using RNA isolated from KRBP72 RNAi and three complemented cell lines in the absence or presence of doxy for 3 days. Reverse transcription was carried out with an oligo(dT) primer, and PCR was performed with primers specific to the 5′ and 3′ ends of the A6 mRNA to amplify the entire population of A6 mRNAs, including pre-edited, partially edited and fully edited RNA. L, size ladder.

Thus, to further probe RNA-binding and ATPase contribution to KRBP72 function, we complemented the KRBP72 knockdown with endogenously TY-tagged wild type, RNA-binding mutant (RR-AA), or ATPase mutant (K-A) KRBP72, resulting in cells expressing primarily wild-type or mutant proteins at levels approximating those of endogenous KRBP72 (Figure 5A). We first confirmed that the RR to AA substitutions at amino acids 198/199 decreased KRBP72 binding to A6 mRNA *in vivo*, as previous RNA-binding assays were performed *in vitro* on GC-rich oligos and oligos corresponding to short regions of some mitochondrial mRNAs (23). To test this, we performed RIPs from cells complemented with wild type (WT) or either of the mutant KRBP72 proteins and measured enrichment of total A6 mRNA in KRBP72 pulldowns compared to control beads. As shown in Figure 5B, the RR-AA mutant exhibited diminished A6 mRNA binding as expected, whereas RNA binding in the K-A mutant exhibited significantly greater binding than the RR-AA mutant in this assay. We next tested the effects of KRBP72 mutations on A6 mRNA editing. Complementation with TY-tagged wild-type protein restored edited A6 mRNA levels to 75% of levels in wild-type cells as measured by qRT-PCR (Figure 5C). Surprisingly, complementation with the RNA-binding mutant similarly restored edited A6 mRNA levels. By contrast, cells complemented with the ATPase mutant failed to restore edited A6 mRNA levels above those seen in KRBP72 knockdown cells. To confirm these results, we performed full gene PCRs to visualize pausing in the ES121-126 region (Figure 5D). Consistent with qRT-PCR results, WT and RNA-binding mutant KRBP72 partially restored the wild-type editing pattern and decreased pausing at ES121-126, while the ATPase mutant failed to do so. From these data, we conclude that KRBP72’s ATPase activity is critical in facilitating A6 mRNA editing *in vivo*, while its stable RNA-binding activity is dispensable.

### KRBP72 exhibits RNase-sensitive and RNase-insensitive interactions with RESC proteins

**Figure 6.**
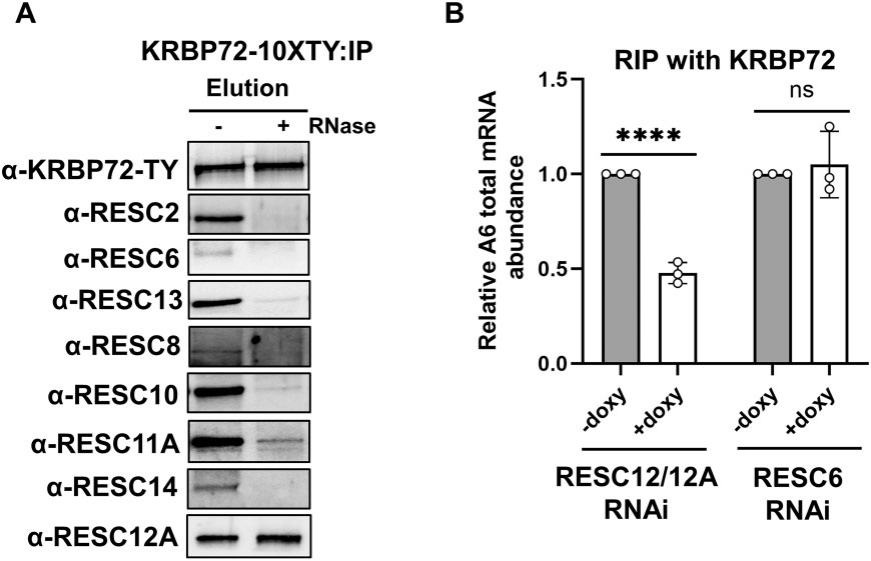
RESC factor association with KRBP72 and influence on KRBP72 RNA binding. (**A**) KRBP72-10XTY was immunoprecipitated with α-TY antibodies from untreated or RNase-treated whole cell extracts. After normalization of KRBP72 levels, immunoprecipitates were immunoblotted with antibodies against various RESC proteins. This dataset corresponds to one of two biological replicate experiments, with the exception of RESC12A, which was performed in biological triplicate. (**B**) RIP comparing total A6 RNA immunoprecipitated with α-KRBP72 antibodies from uninduced (−doxy) or induced (+doxy) RESC12/12A or RESC6 RNAi cells. Relative abundance in RESC12/12A RNAi and RESC6 RNAi represents the A6 RNA levels detected in the immunoprecipitate from induced cells compared to the immunopreciptate from uninduced cells. RNA levels were standardized against 18S rRNA, and numbers represent the mean of three biological replicates, each with three technical replicates. Significance was evaluated using Student’s *t*-test. ns, not significant; ****P < 0.0001.

KRBP72 was previously reported to exhibit RNase-sensitive interactions with several RESC components (23). In light of our increased understanding of RESC organization (17) and expanded antibody panel (13), we wanted to further probe the KRBP72-RESC interaction to gain insight into KRBP72 function. To this end, we endogenously tagged KRBP72 with a TY tag at the C-terminus and conducted immunoprecipitation using anti-TY antibodies in the presence and absence of RNase (Figure 6A). Our findings align with those of Shaw *et al.* (23), confirming that KRBP72 interacts with RESC2, RESC6 and RESC13 in an RNase-sensitive manner. We also identified RNase-sensitive interactions between KRBP72 and RESC8, RESC10, RESC11A and RESC14. Remarkably, however, association of KRPB72 with RESC12A was entirely unperturbed by RNase treatment, suggesting an RNA-independent KRBP72-RESC12A interaction.

### RESC12/12A facilitate interaction of KRBP72 with A6 RNA

To understand the functional significance of the KRBP72-RESC12A interaction, we first verified that RESC12A’s association with other RESC components is not impacted by KRBP72 knockdown (Supplementary Figure S8). Next, we asked how RESC12A might influence KRBP72 function. Given that KRBP72’s stable RNA-binding activity is not required for A6 mRNA editing (Figure 5C) and RESC12A itself is an RNA-binding protein (34), we asked whether RESC12A might contribute to KRBP72’s interaction with A6 mRNA. To do this, we used a published cell line in which RESC12A and the paralogous RESC12 were subjected to doxy-induced RNAi for 3 days (34). After confirming the knockdown and establishing that total A6 mRNA was enriched compared to control beads in these cells (Supplementary Figure S9A and B), we compared KRBP72 association with total A6 mRNA in the presence and absence of RESC12/12A by RIP using anti-KRBP72 antibodies. Following immunoprecipitation, we isolated RNA from anti-KRBP72 and control beads and quantified total A6 mRNA by qRT-PCR (13,14). Figure 6B shows that depletion of RESC12/12A leads to more than 2-fold reduction in the ability of KRBP72 to stably associate with total A6 mRNA compared to uninduced cells. To determine if this effect is specific to RESC12/12A or extends to other RESC factors, we performed the analogous experiment with RESC6 RNAi cells uninduced or doxy-induced for 3 days (40) (Supplementary Figure S9 and Figure 6B). RESC6 depletion had no effect on the ability of KRBP72 to associate with A6 mRNA. Thus, we conclude that RESC12/12A plays a crucial role in facilitating the interaction between A6 mRNA and KRBP72.

## Discussion

In this study, we elucidated the pivotal role of KRBP72 in the editing of A6 mRNA in *T. brucei*. We observed distinct effects of KRBP72 knockdown in PCF and BSF life cycle stages. While fully edited A6 mRNA levels are decreased in both knockdowns, accumulation of a specific partially edited mRNA species was observed only in PCF. Even in uninduced cells, the accumulating partially edited A6 mRNAs differed between the two life cycle stages, despite the presence of constant KRBP72 protein levels. The distinctions in partially edited mRNA populations may be attributed to the different environmental conditions, as PCF cells grow at 27°C while BSF cells grow at 37°C, potentially leading to differences in the A6 RNA structure at these temperatures.

**Figure 7.**
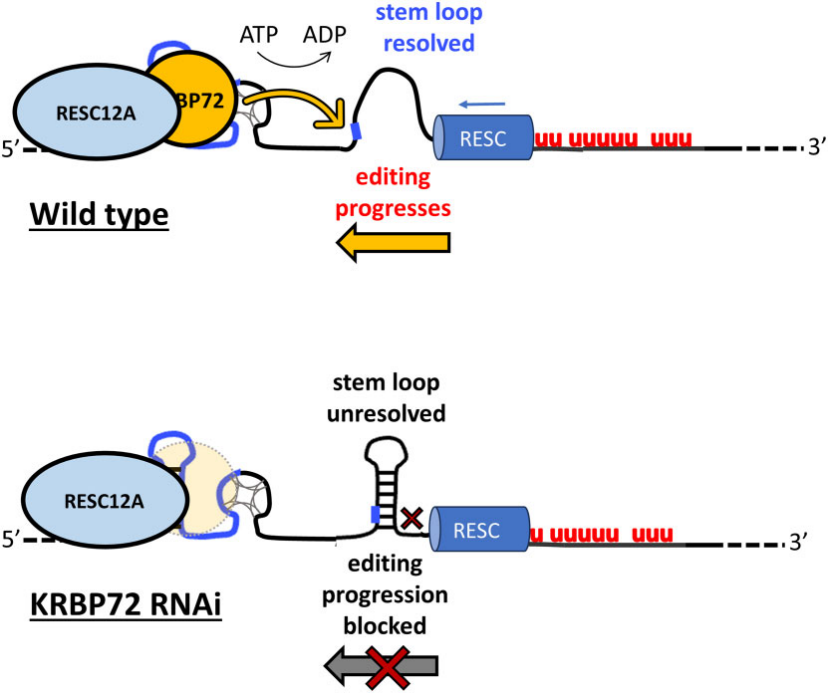
Working model of KRBP72 action. In wild-type cells, KRBP72 associates with RESC12A to facilitate editing progression through a predicted stem loop region in a manner dependent on KRBP72 ATPase activity. Upon KRBP72 RNAi, editing pauses in the stem loop region. See text for details.

Indeed, our single nucleotide level analysis of paused A6 mRNAs in PCF KRBP72 knockdowns implicated mRNA structure as a critical factor in KRBP72 function. KRBP72 depletion disrupts the progression, rather than the initiation, of RNA editing, resulting in a major pause at a stem-loop structure within the pre-edited region of A6 mRNA as determined by SHAPE and RT-stop analysis under differing ionic conditions (54,55) (Figure 7). We also considered that pausing actually occurs, not in a completely pre-edited RNA, but rather in a partially edited molecule with a sequence somewhat different from that shown in Figures 3D and 4B. Structure prediction of an RNA that is fully edited 3′ of ES 124 and pre-edited 5′ of this site using Vienna RNA suggests a different structure in which three of the G-C basepairs located at the base of the stem in the pre-edited mRNA now interrupt a large single stranded region (Supplementary Figure S10). Biochemical validation of this structure may be useful in the future. However, the predicted sequence presumes that this mRNA is created *de novo* with this partially edited sequence, which is fully available for folding. Rather, the mRNA is transcribed as a pre-edited mRNA that presumably folds similarly to the structure determined by Leeder *et al.* (54,55) (Figure 4B). Progressive editing from 3′ to 5′ may lead to local structure changes; however, U-rich edited sequences at the 3′ end of the mRNA may not disrupt the stable stem loops and G-quadruplexes further 5′ that are already formed in the pre-edited region of the molecule (19). Thus, we find it most likely that KRBP72 facilitates progression through a stem loop as shown in Figures 3D and 4B. Regardless of the precise structure, however, KRBP72 clearly promotes editing through a structural element comprised of a series of G-C basepairs. These findings add to a growing body of data that implicate mRNA structure as an important determinant in U-indel editing progression, including creation of elements that regulate editing during *T. brucei* development (19,20).

KRBP72 occupies one of the basal branches of the ABC superfamily of ATPases, a ubiquitous protein family, one function of which is modulating the dynamics of nucleic acids and nucleoprotein complexes (23,59). For example, Rad50, a component of the MRE11/Rad50 complex, undergoes an ATP hydrolysis-dependent conformational change that leads to DNA melting during DNA stranded break repair in *Methanococcus jannaschii* (60). MutS2 in *Bacillus subtillis* binds stalled ribosomes and acts in ribosomal rescue by mediating ATP-driven ribosomal subunit splitting (61). In yeast, eukaryotic elongation factor 3 facilitates late stages in tRNA translocation during translation by favoring the transition toward the non‐rotated conformation of the ribosome and by influencing the conformation of the L1 stalk (62). Here, we established that the ATPase activity of KRBP72 is indispensable for its role in A6 mRNA editing, as a KRBP72 protein with mutations in the ATPase domain fails to rescue editing in the knockdown. Because editing pauses at the base of a stem loop (ES121-126) in these cells, the simplest explanation is that KRBP72 ATPase activity is used to unwind the stem loop to allow editing to progress through this region of the transcript (Figure 7). However, we cannot rule out other functions of its ATPase activity such reorganization of the editing machinery in a manner that allows progression through the stem loop or removal of stalled editing complexes. Futures studies to distinguish these possibilities will be of interest.

The N-terminal domain of KRBP72 reveals the RAGNYA fold, a fold observed in several other nucleic acid-binding contexts (59). The KRBP72 dimer harbors a unique pore formed by its dimeric N-terminal domains, two arginine residues of which are critical for its RNA-binding activity *in vitro* (23). Our mutational study confirms that these two arginine residues are crucial for binding A6 mRNA *in vivo*, as RR-AA mutants exhibited a greater than 2-fold decrease RNA-binding activity compared to WT KRBP72 in RIP experiments. eCLAP confirmed the interaction of KRBP72 with pre-edited regions of A6 mRNA *in vivo*, with some binding to the stem loop that confers pausing and a much stronger binding peak 5′ of this region around the nearby G-quadruplex and an upstream stem loop. These binding characteristics suggest two possibilities. KRBP72 may directly bind to the stem loop, as indicated by the two eCLAP sites within the stem, to maintain the proper RNA structure for editing to proceed through this region. Alternatively, KRBP72 may initially bind to the G-quadruplex, which in turn is configured in 3D space so as to allow the protein to access the stem loop (Figure 7). Editing at the pause sites between A6 mRNA ES121-126 is directed by the middle of the guiding region of A6 gRNA12 (43). KRBP72-mediated stem loop disruption presumably allows this gRNA to access ES121-126 in A6 mRNA. However, we cannot rule out a direct effect of KRBP72 on the structure of A6 gRNA12, which in turn facilitates editing in this region.

Surprisingly, the intrinsic KRBP72 RNA-binding activity was not required for its action in progression of A6 mRNA editing, as the RR-AA mutant was able to restore edited A6 mRNA levels to the same extent as did wild-type KRBP72. To address this apparent paradox, we sought KRBP72 partners that might contribute to its *in vivo* RNA-binding activity. Of numerous RESC proteins tested, we found that only the RNA-binding protein, RESC12A, interacts with KRBP72 in an RNase-insensitive manner. RNAi-mediated depletion of RESC12A, but not that of another RESC protein, RESC6, led to a substantial decrease in the KRBP72-RNA interaction. Thus, RESC12A appears to facilitate binding of KRBP72 to A6 mRNA and/or stabilize its binding there. These findings provide insight not only into KRBP72 function, but also into the role of RESC12A in editing. RESC12A is considered a component of the REMC (RNA Editing Mediator Complex) submodule of RESC, along with RESC12, RESC13 and RESC11 (5). However, single nucleotide level analysis of edited mRNA populations indicates that, while RESC13 and RESC11 generally share the same role in editing, the specific function of RESC12A differs markedly from that of the other two proteins (5). In addition, RESC12A was not visible on recent cryo-EM images of RESC, whereas the positions of RESC13 and RESC11 at the pre-edited mRNA end of the complex were clear (17). Thus, the function(s) of RESC12A in editing appear distinct from those of other REMC proteins. In eCLAP experiments, RESC12A showed promiscuous RNA binding in terms of both the range of transcripts bound and binding along the entire lengths of many pan-edited mRNAs, leading the authors to suggest that it flags regions that need to be edited on these mRNAs (63). On minimally edited mRNAs, including CYb, RESC12A binding correlated to that of the auxiliary factor, MRP1/2, which promotes CYb mRNA editing (64). Interestingly, biochemical experiments showed that knockdown of RESC12A leads to a decrease in RNA binding of auxiliary factors MRP1 and KRGG1, suggesting that RESC12A recruits these proteins to pre-edited mRNAs (63,64). Together with our data showing that RESC12A is required for maximal KRBP72 RNA binding, these data suggests that a conserved function of RESC12A is recruitment of auxiliary editing factors. Overall, with regard to KRBP72, we show that both its intrinsic RNA-binding activity and RESC12A-mediated RNA-binding activity contribute to its association with A6 mRNA *in vivo*. However, only the RESC12A-mediated binding is critical for A6 editing progression (Figure 7). This distinction suggests differences in the two modes of KRBP72 mRNA binding potentially involving stability, specificity, or resultant modulation of RNA structure.

It is of interest that KRBP72 depletion specifically impacts editing of the A6 transcript. A6 is the only mitochondrially encoded component of the F_0_-F_1_-ATP synthase, which synthesizes ATP in the PCF stage of the *T. brucei* life cycle. In BSF, this complex operates in reverse mode, hydrolyzing ATP to maintain mitochondrial membrane potential (65). Due to the constant need for a functional ATP synthase/ATPase complex, the A6 transcript undergoes extensive and continuous editing throughout the entire life cycle of the parasite (66). Mounting evidence suggests that editing of this transcript is particularly dependent on auxiliary factors, which may act in concert to ensure the production of translatable A6 mRNA. In addition to the requirement for KRBP72 reported here, Carnes *et al.* (26) showed that KRGG1 is essential for A6 mRNA editing in BSF *T. brucei* as conditional knockout caused pausing at a site distinct from that observed here in PCF KRBP72, with no detrimental effects on other transcripts. A6 mRNA is also the only transcript whose editing in PCF depends on the KREH1 RNA helicase (31). KREH1 knockout in the PCF led to the inability to properly utilize the initiator gRNA as well as numerous pauses throughout the A6 mRNA, likely also due to impaired RNA structural rearrangements (31). Another helicase, KREH2, which is considered part of the editing holoenzyme, was reported to differentially control non-canonical editing and putative repressive structures in A6 mRNA via a novel proposed ‘bifunctional’ gRNA in A6 mRNA (19), highlighting an additional mechanism for control of editing of this transcript. Additionally, three of the above-mentioned factors, KRBP72, KREH1 and KREH2, are ATP-binding proteins, suggesting they could serve as sensors of the ATP/ADP (adenosine diphosphate) ratio in the mitochondria to regulate levels of A6 mRNA editing in one or both life cycle stage. Such a function would be analogous to that of the ABC family ATPase, EttA, which stalls peptide bond formation and causes termination when it is predominantly bound to ADP; it allows peptide bond formation only when ATP concentrations are sufficient to occupy its active site (67,68). KRBP72 exhibits distinct conformational states influenced by nucleotide binding. The open state, which is stabilized by ADP binding, allows RNA to bind within the pore (23,59), which may be a prerequisite for promoting editing through the inhibitory stem loop in the A6 transcript.

In summary, this study provides valuable insights into the role of the KRBP72 ATPase in A6 RNA editing, and increases our understanding of editing auxiliary factors and their functional interactions with the RESC component of the editing holoenzyme. The widespread KRBP72 RNA-binding sites identified our eCLAP analysis suggest that KRBP72 has additional roles in both the mitochondria and cytoplasm. Future studies will likely clarify KRBP72 function beyond U-indel RNA editing and may reveal links between editing and other cellular processes.

## Supplementary Material

gkae1153_Supplemental_Files

## Data Availability

RNA seq data are available at the Sequence Read Archive under accession numbers PRJNA1133815 and eCLAP data available under GEO accession numbers GSE272305.
