# Supplementary material for: KRBP72 facilitates ATPase-dependent editing progression through a structural roadblock in mitochondrial A6 mRNA
Source: Nucleic Acids Res. 2024 Dec 3;53(2):gkae1153. doi: 10.1093/nar/gkae1153 (PMC11754742; doi:10.1093/nar/gkae1153)
Supplement: gkae1153_Supplemental_Files [file gkae1153_supplemental_files.zip › KRBP72 SUPPLEMENTAL FIGURES Rev2 Oct25 LR.pdf]

Supplementary Materials for

KRBP72 facilitates ATPase-dependent editing progression through a structural roadblock in mitochondrial A6 mRNA

Ashutosh P. Dubey[1], Brianna L. Tylec[1], Soon Yi[2,3], Frank A. Tedeschi[2,3], Joseph T. Smith[1], and Laurie K. Read[1]*

[1]Department of Microbiology and Immunology, University at Buffalo Jacobs School of Medicine and Biomedical Sciences, Buffalo, NY 14203; [2]Center for RNA Science and Therapeutics, School of Medicine, Case Western Reserve University, Cleveland, OH 44106 [3]Department of Biochemistry, School of Medicine, Case Western Reserve University, Cleveland, OH 44106.

The file includes: Tables S1, S4, and S5; Figs. S1 to S10 with legends

Other Supplementary Material for this manuscript includes the following: Tables S2 and S3.

*To whom correspondence should be directed:
Laurie K. Read
lread@buffalo.edu
(716)829-3307

Table S1. Primers used in this study

Primers	Sequence (5'-3')
KRBP72_5'-F	CGCGGATCCCACAGATTTGGCGGCGGCGAA
KRBP72_5'-R	CCCAAGCTTAATAACCATGATGGAGGAAAC
KRBP72_SD-F	TCACCTCAGCAACAAGGCACATAGAGATTGGTGCTGCCCTCAATCGTTTGCG TACACTCCGGACCGTTACTGCGAAAAGAGGTTCTGGTAGTGGTTCC
KRBP72_SD-R	GTACGCACGTGTGTGCGTGTGAGCGCATAGTGGACCAATAAATATTATCATG GAGACAGTGCACACATCTCTTTATCCTTCCAATTTGAGAGACCTGTGC
RESC12_SD-F	TTACGGCGGAACTGGCGCGCCGATTTGGTGGGGGACAGGAGCAGCAAATGGC GCAGAAGATGTTAGCGAAGGTATTTTGTGGTTCTGGTAGTGGTTCC
RESC12_SD-R	GTTATGCACGCATTACCCACGGTCCAGTACCTTTGAGGCTCCTTAATTTGCC TCTTTAGTTGCTCATCCAGCCTTTCGGACCAATTTGAGAGACCTGTG
pET28a_KRBP72-F	CCCAAGCTTATGTACCATCGTGGCTACG
pET28a_KRBP72-R	CCGCTCGAGTCACAGATCCTCTTCTGAGA
A6_3'-F	AAAACACCCATTTTTAGGAGG
A6_3'-R	CTATATTTGTCTTATTCTATAACTCC
A6_Central-F	AGAAAAGTAGGGGAATTTTG
A6_Central-R	TGGCGGCAAATAAATCC

Table S4. eCLAP mapping to whole *T. brucei* genome

	Sample 1	Sample 2	Sample 3
Total Reads	108,504,527 (100%)	120,918,076 (100%)	135,873,925 (100%)
Aligned 0 times	22,929,670 (21.2%)	24,440,901 (20.2%)	18,979,106 (14.0%)
Aligned 1 time	10,444,337 (**9.63%**)	14,250,436 (**11.8%**)	23,439,694 (**17.3%**)
Aligned >1 times	75,130,520 (69.2%)	82,226,739 (68.0%)	93,455,125 (68.8%)
Overall %	78.9%	79.8%	86.0%
Peaks Called	**61025 high confidence peaks**		

Table S5. eCLAP mapping to *T. brucei* mitochondrial genome

	Sample 1	Sample 2	Sample 3
Total Reads	108,504,527 (100%)	120,918,076 (100%)	135,873,925 (100%)
Aligned 0 times	108,130,548 (99.6%)	119,990,963 (99.2%)	135,427,497 (99.67%)
Aligned 1 time	365,912 (**0.34%**)	861,471 (**0.71%**)	436,605 (**0.32%**)
Aligned >1 times	8,067 (0.01%)	65,642 (0.05%)	9,823 (0.01%)
Overall %	0.34%	0.77%	0.33%
Peaks Called	**40 high confidence peaks**		

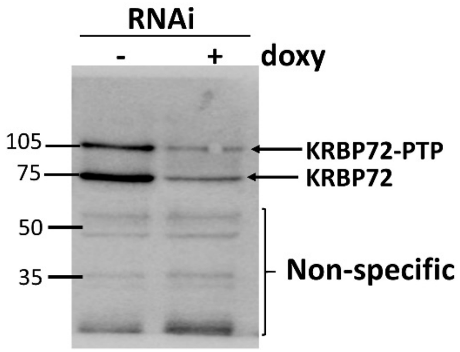

Figure S1: Validation of KRBP72 antibody. Western blot showing affinity purified anti-KRBP72 antibody detection of both endogenous KRBP72 and KRBP72-PTP in a cell line harboring both one PTP-tagged allele and doxy regulated KRBP72 RNAi. KRBP72 proteins are the primary reactive bands.

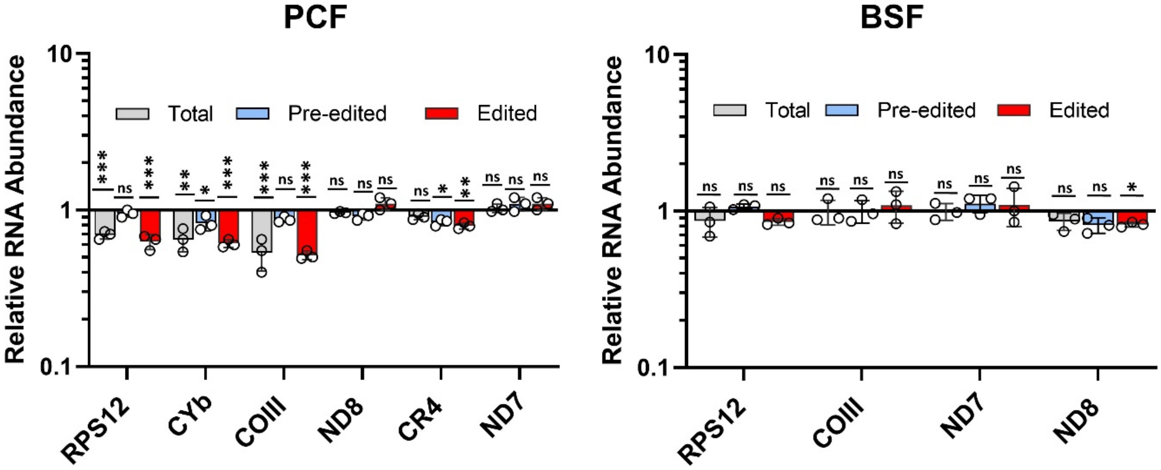

Figure S2: Effect of KRBP72 depletion on *T. brucei* RNA editing in two life cycle stages. RNA was isolated from KRBP72 RNAi cells grown in the absence or presence of doxy for 3 days for PCF cells and 2 days for BSF cells. RNA levels were quantified by qRT-PCR using primer sets specific for pre-edited, fully edited, and total RNAs. Relative RNA abundance represents levels in +doxy (RNAi induced) cells compared to levels in -doxy (uninduced) cells, normalized to 18S rRNA levels. Three biological replicates were performed, each with three technical replicates. Significance was evaluated using Student's t-test. ns, not significant; *P<0.05; ** < 0.01; *** P < 0.001.

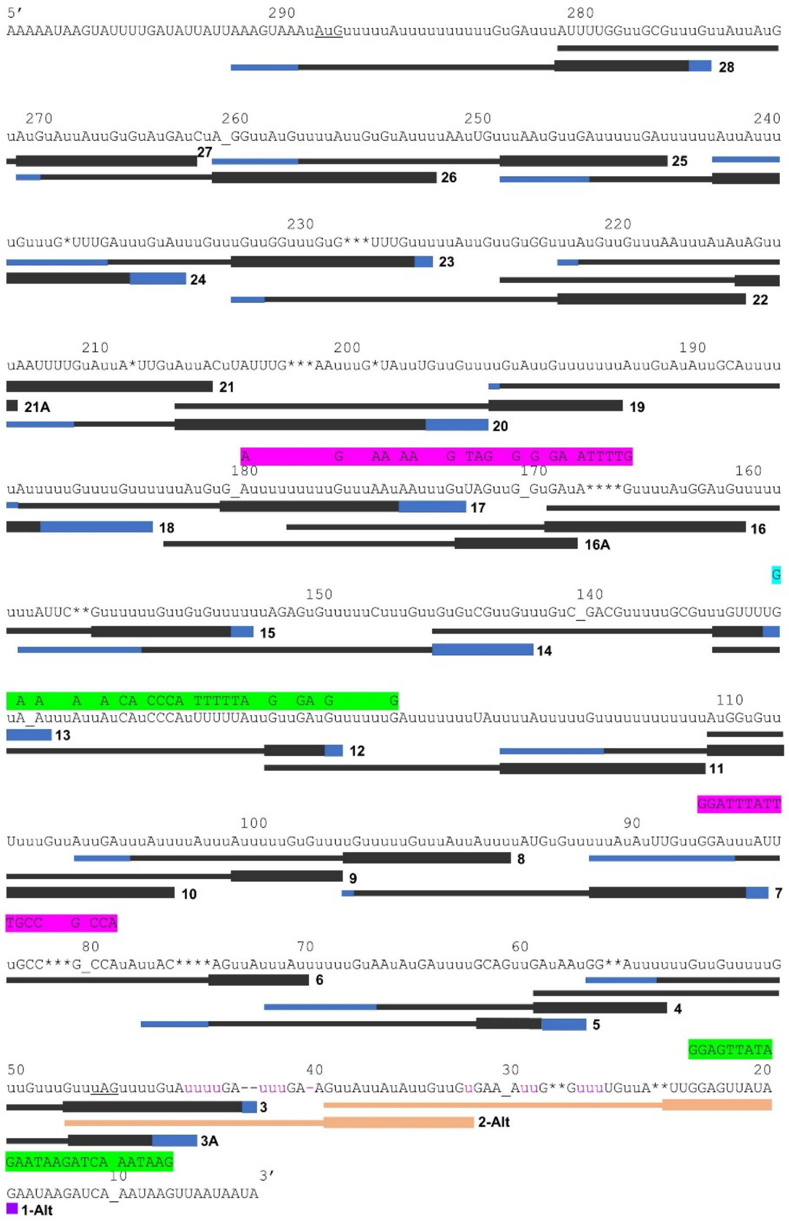

Figure S3: A6 edited mRNA sequence. Bars below the sequence indicate gRNAs, numbered 3′ to 5′. gRNA anchor regions are depicted with bold lines. Blue regions at the 5′ and 3′ ends represent variation in gRNA lengths within a gRNA class. Start and stop codons are underlined; additional underscores are shown for clarity in stretches of unedited sequence to align numbers with the correct editing sites. A green-colored background indicates the primer used for 3' region amplification, while a purple-colored background indicates the primer used for central region amplification.

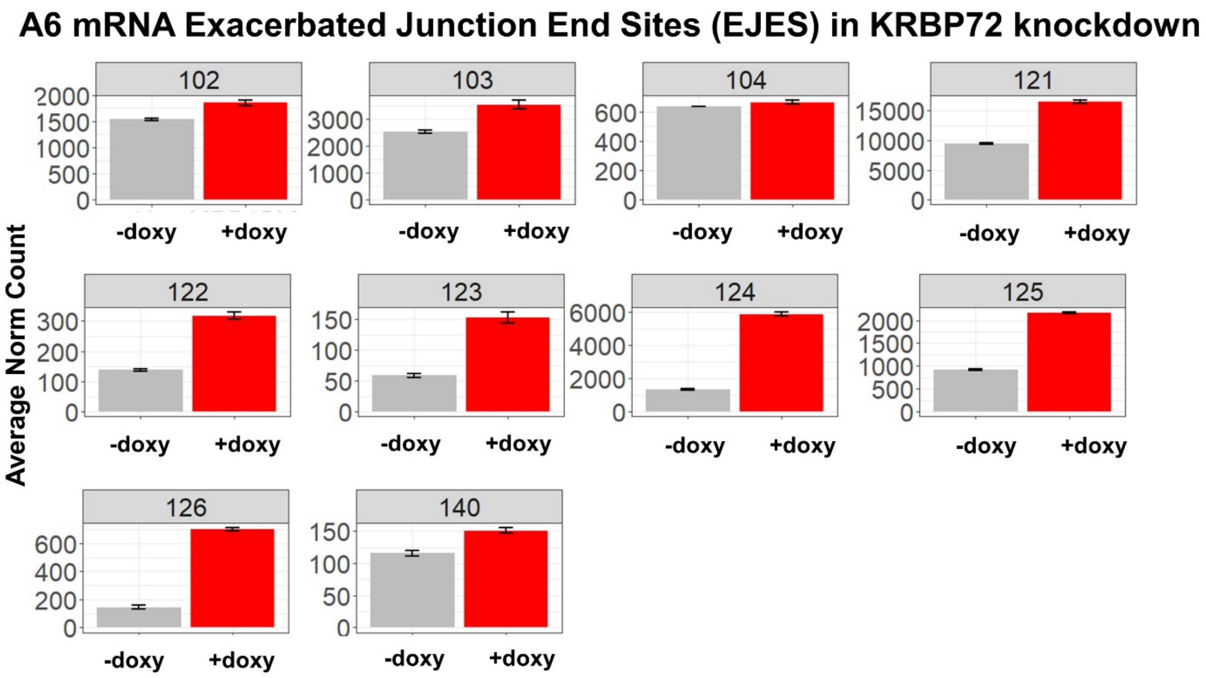

Figure S4: Normalized read counts for those JES determined to be EJES in induced KRBP72 knockdown vs. uninduced cell controls. Any JES for which the number of reads is significantly higher (padj < 0.05) in the KRBP72 depleted condition than in the KRBP72 replete condition for both KRBP72 knockdown replicates is considered an EJES.

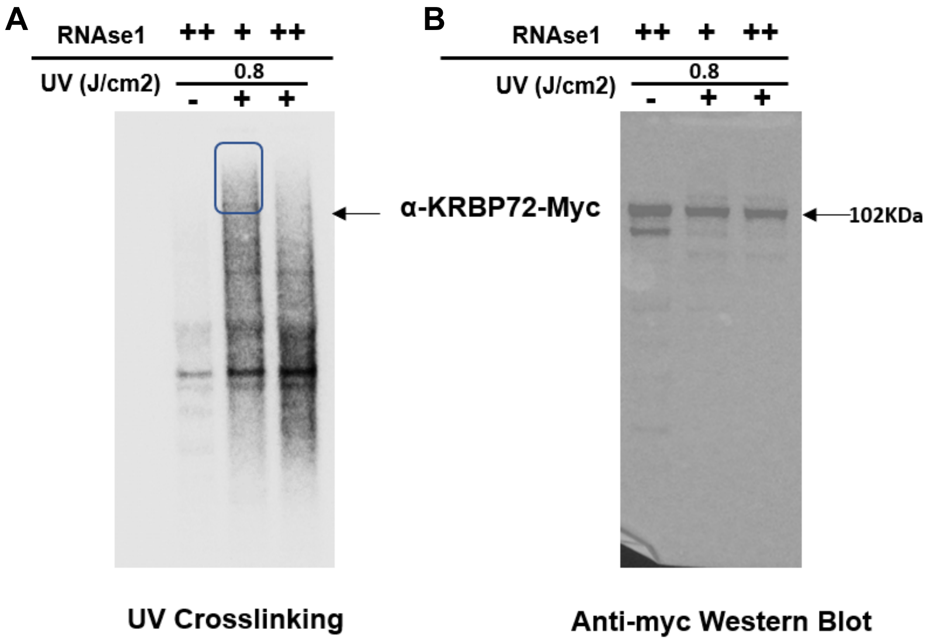

Figure S5: KRBP72 eCLAP. **(A)** Purification of UV-cross-linked RNA-KRBP72-MHT complex. After Ni-NTA-His affinity purification, the ^{32}P-labeled complexes were monitored by autoradiography. The optimal 0.8-J/cm2 UV radiant fluence was used to *in vivo* cross-link RNA to proteins. Non-UV-cross-linked KRBP72-MHT-tagged (lane 1) yielded no signal. The high-RNase I treatment of UV-cross-linked KRBP72 (lane 3) showed a band at ~102-kDa size. The low-RNase treatment (boxed region in lane 2) was used to prepare the KRBP72 eCLAP libraries. **(B)** KRBP72 IP was confirmed using Myc antibody. Three independent replicates were performed for preparation of the eCLAP library.

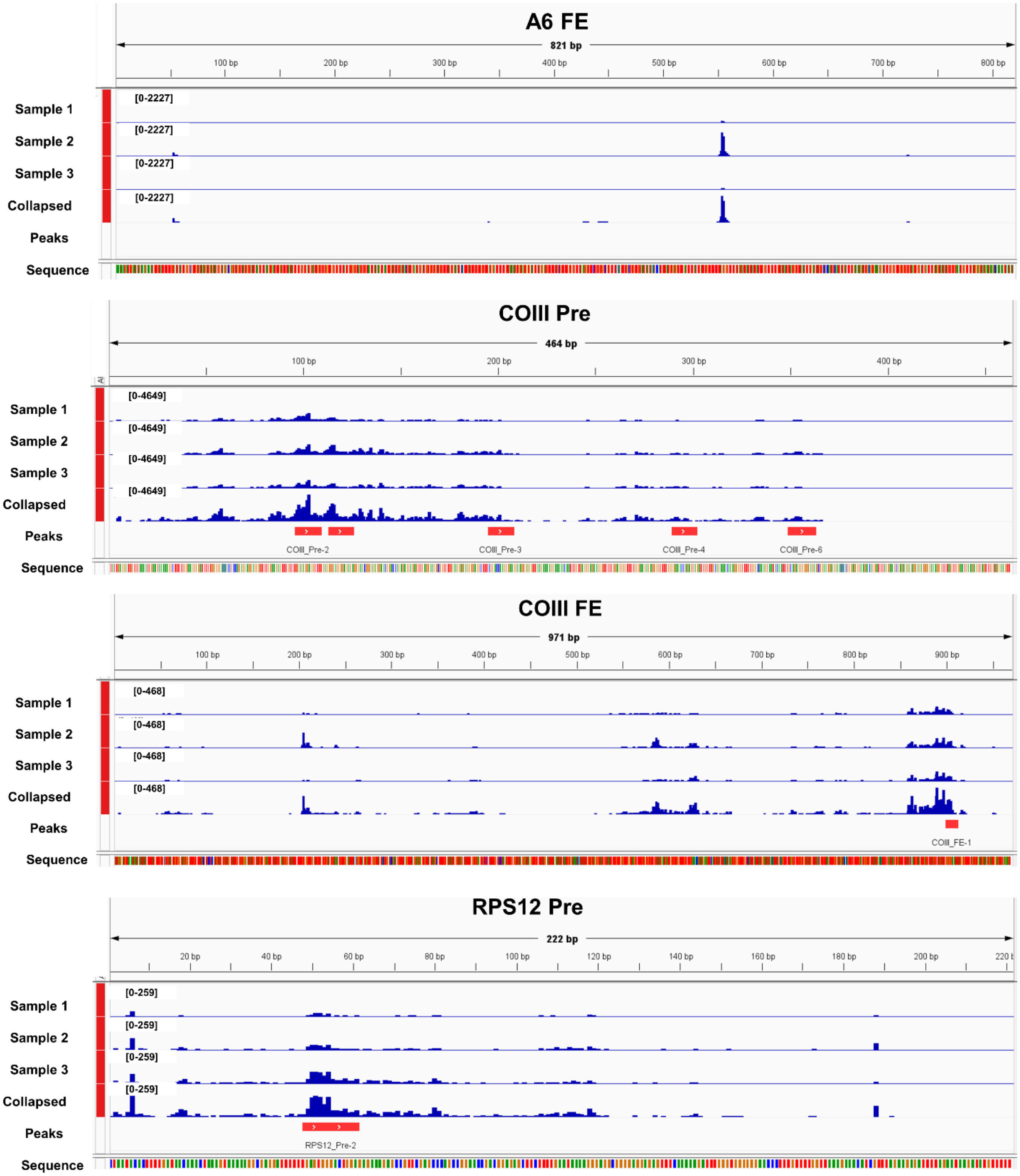
A6 FE
821 bp
100 bp
200 bp
300 bp
400 bp
500 bp
600 bp
700 bp
800 bp
Sample 1
Sample 2
Sample 3
Collapsed
Peaks
Sequence
[0-2227]
[0-2227]
[0-2227]
[0-2227]
COIII Pre
464 bp
100 bp
200 bp
300 bp
400 bp
Sample 1
Sample 2
Sample 3
Collapsed
Peaks
Sequence
[0-4649]
[0-4649]
[0-4649]
[0-4649]
COIII_Pre-2
COIII_Pre-3
COIII_Pre-4
COIII_Pre-6
COIII FE
971 bp
100 bp
200 bp
300 bp
400 bp
500 bp
600 bp
700 bp
800 bp
900 bp
Sample 1
Sample 2
Sample 3
Collapsed
Peaks
Sequence
[0-468]
[0-468]
[0-468]
[0-468]
COIII_FE-1
RPS12 Pre
222 bp
20 bp
40 bp
60 bp
80 bp
100 bp
120 bp
140 bp
160 bp
180 bp
200 bp
Sample 1
Sample 2
Sample 3
Collapsed
Peaks
Sequence
[0-259]
[0-259]
[0-259]
[0-259]
RPS12_Pre-2

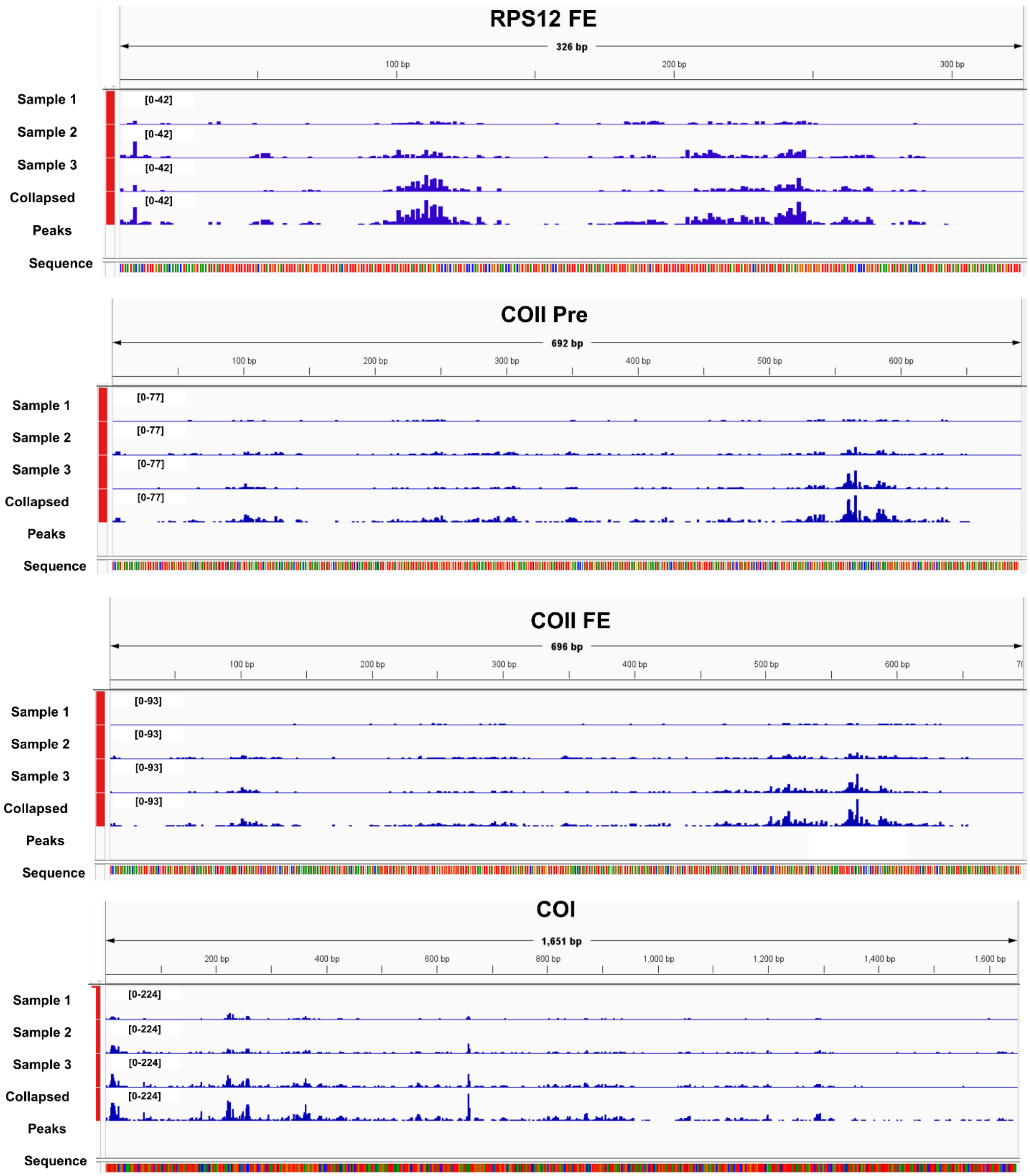

RPS12 FE
326 bp
100 bp
200 bp
300 bp
Sample 1
Sample 2
Sample 3
Collapsed
Peaks
Sequence
[0-42]
[0-42]
[0-42]
[0-42]
COII Pre
692 bp
100 bp
200 bp
300 bp
400 bp
500 bp
600 bp
Sample 1
Sample 2
Sample 3
Collapsed
Peaks
Sequence
[0-77]
[0-77]
[0-77]
[0-77]
COII FE
696 bp
100 bp
200 bp
300 bp
400 bp
500 bp
600 bp
Sample 1
Sample 2
Sample 3
Collapsed
Peaks
Sequence
[0-93]
[0-93]
[0-93]
[0-93]
COI
1,651 bp
200 bp
400 bp
600 bp
800 bp
1,000 bp
1,200 bp
1,400 bp
1,600 bp
Sample 1
Sample 2
Sample 3
Collapsed
Peaks
Sequence
[0-224]
[0-224]
[0-224]
[0-224]

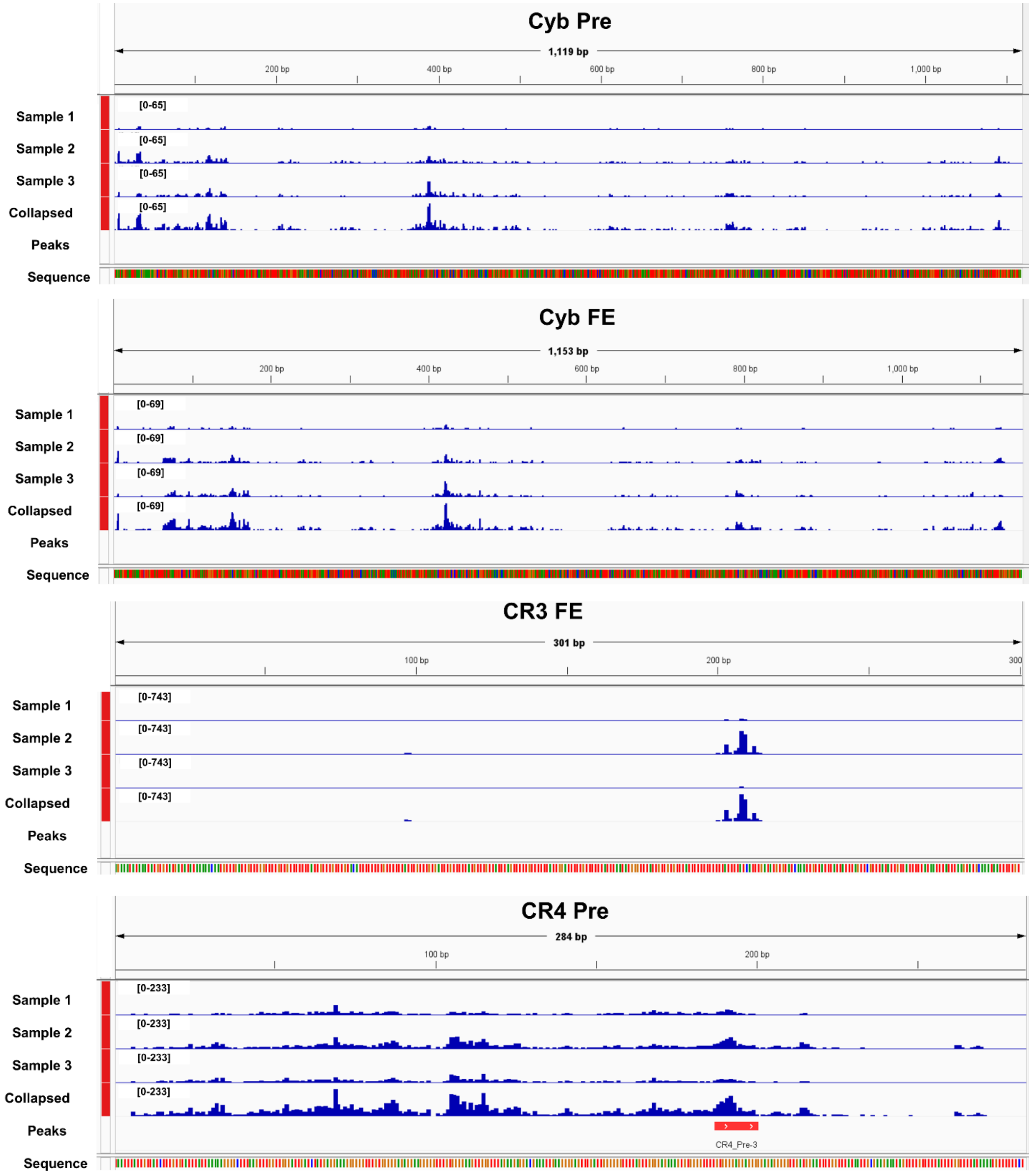

Cyb Pre
1,119 bp
200 bp
400 bp
600 bp
800 bp
1,000 bp
Sample 1
Sample 2
Sample 3
Collapsed
Peaks
Sequence
[0-65]
Cyb FE
1,153 bp
200 bp
400 bp
600 bp
800 bp
1,000 bp
Sample 1
Sample 2
Sample 3
Collapsed
Peaks
Sequence
[0-69]
CR3 FE
301 bp
100 bp
200 bp
300
Sample 1
Sample 2
Sample 3
Collapsed
Peaks
Sequence
[0-743]
CR4 Pre
284 bp
100 bp
200 bp
Sample 1
Sample 2
Sample 3
Collapsed
Peaks
Sequence
[0-233]
CR4_Pre-3

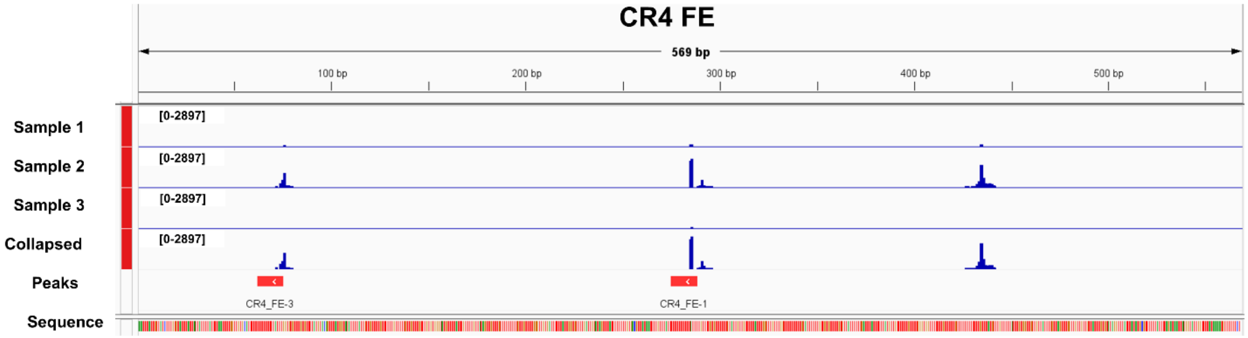
CR4 FE
569 bp
100 bp
200 bp
300 bp
400 bp
500 bp
Sample 1
[0-2897]
Sample 2
[0-2897]
Sample 3
[0-2897]
Collapsed
[0-2897]
Peaks
CR4_FE-3
CR4_FE-1
Sequence

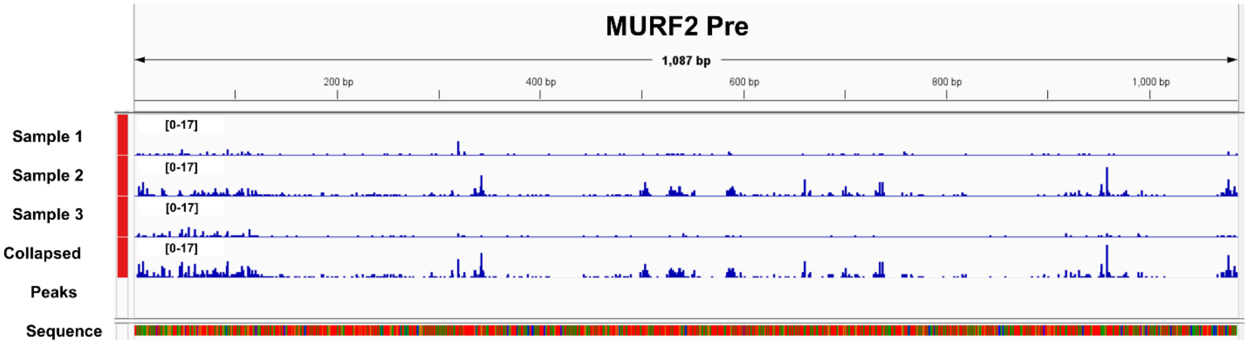
MURF2 Pre
1,087 bp
200 bp
400 bp
600 bp
800 bp
1,000 bp
Sample 1
[0-17]
Sample 2
[0-17]
Sample 3
[0-17]
Collapsed
[0-17]
Peaks
Sequence

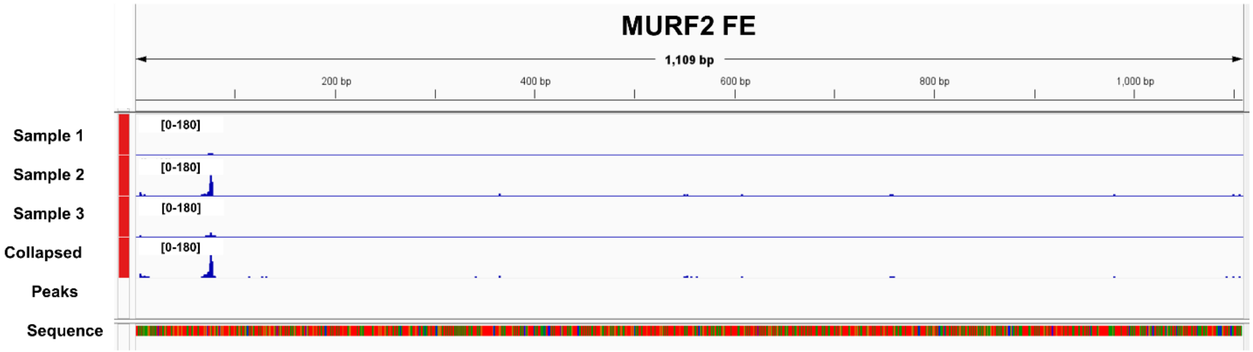
MURF2 FE
1,109 bp
200 bp
400 bp
600 bp
800 bp
1,000 bp
Sample 1
[0-180]
Sample 2
[0-180]
Sample 3
[0-180]
Collapsed
[0-180]
Peaks
Sequence

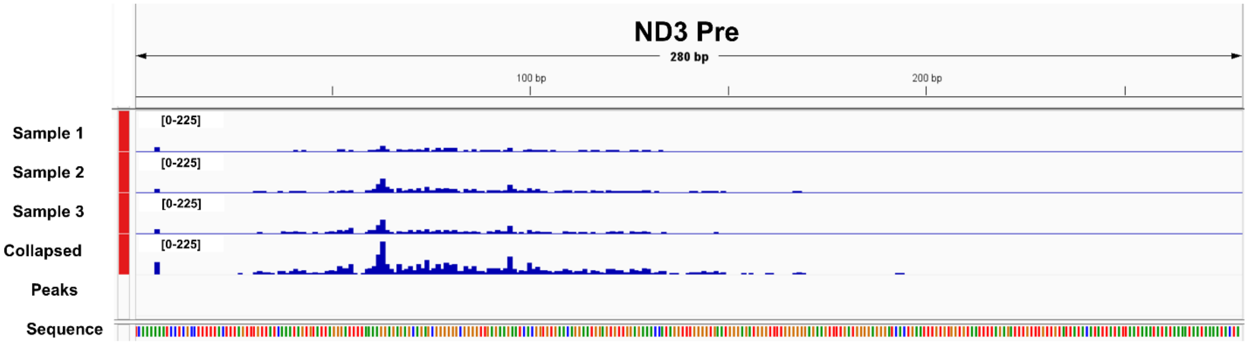
ND3 Pre
280 bp
100 bp
200 bp
Sample 1
[0-225]
Sample 2
[0-225]
Sample 3
[0-225]
Collapsed
[0-225]
Peaks
Sequence

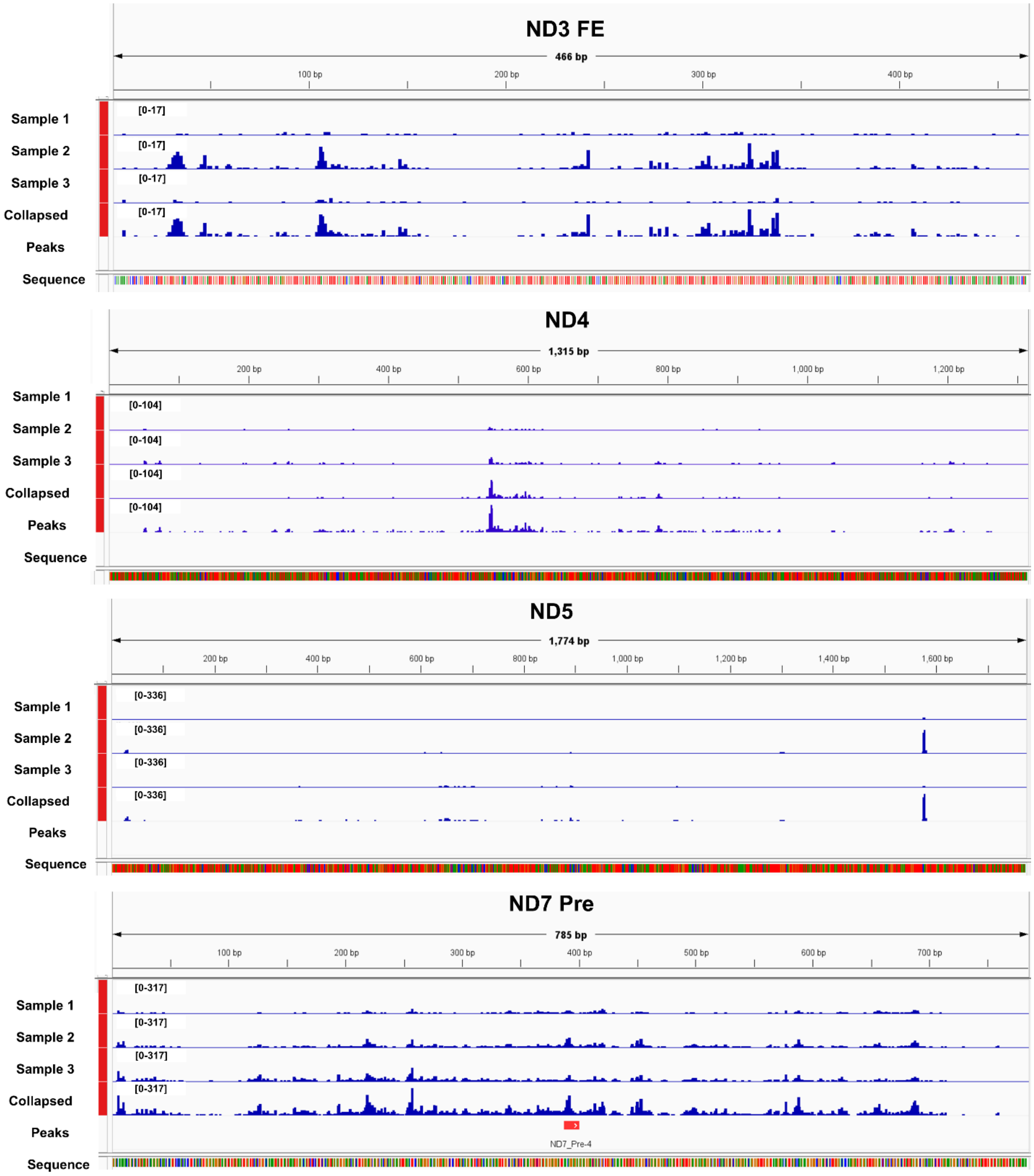
ND3 FE
466 bp
100 bp
200 bp
300 bp
400 bp
Sample 1
Sample 2
Sample 3
Collapsed
Peaks
Sequence
[0-17]
[0-17]
[0-17]
[0-17]
ND4
1,315 bp
200 bp
400 bp
600 bp
800 bp
1,000 bp
1,200 bp
Sample 1
Sample 2
Sample 3
Collapsed
Peaks
Sequence
[0-104]
[0-104]
[0-104]
[0-104]
ND5
1,774 bp
200 bp
400 bp
600 bp
800 bp
1,000 bp
1,200 bp
1,400 bp
1,600 bp
Sample 1
Sample 2
Sample 3
Collapsed
Peaks
Sequence
[0-336]
[0-336]
[0-336]
[0-336]
ND7 Pre
785 bp
100 bp
200 bp
300 bp
400 bp
500 bp
600 bp
700 bp
Sample 1
Sample 2
Sample 3
Collapsed
Peaks
Sequence
[0-317]
[0-317]
[0-317]
[0-317]
ND7_Pre-4

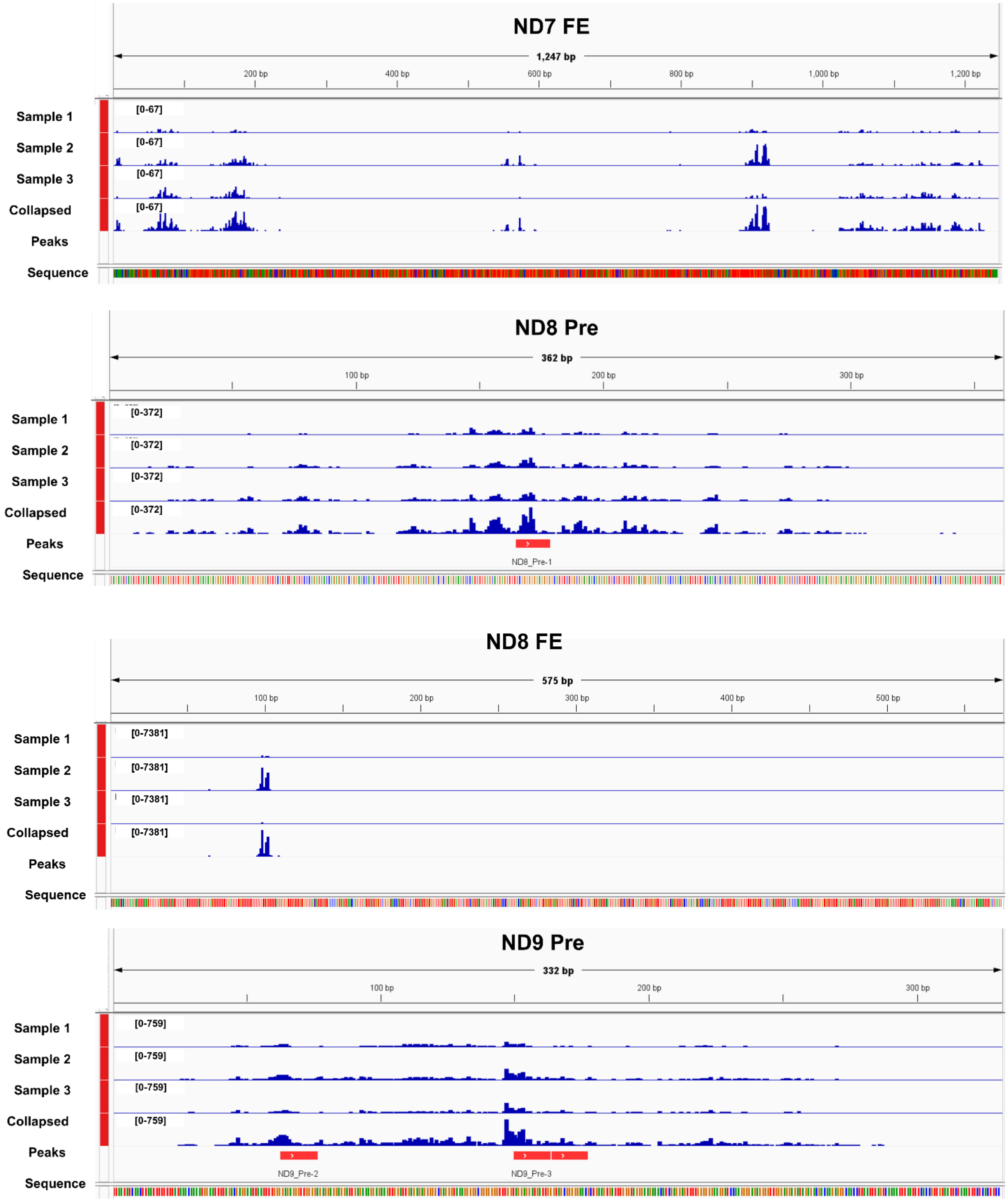

ND7 FE
1,247 bp
200 bp
400 bp
600 bp
800 bp
1,000 bp
1,200 bp
Sample 1
Sample 2
Sample 3
Collapsed
Peaks
Sequence
[0-67]
ND8 Pre
362 bp
100 bp
200 bp
300 bp
[0-372]
ND8_Pre-1
ND8 FE
575 bp
500 bp
[0-7381]
ND9 Pre
332 bp
[0-759]
ND9_Pre-2
ND9_Pre-3

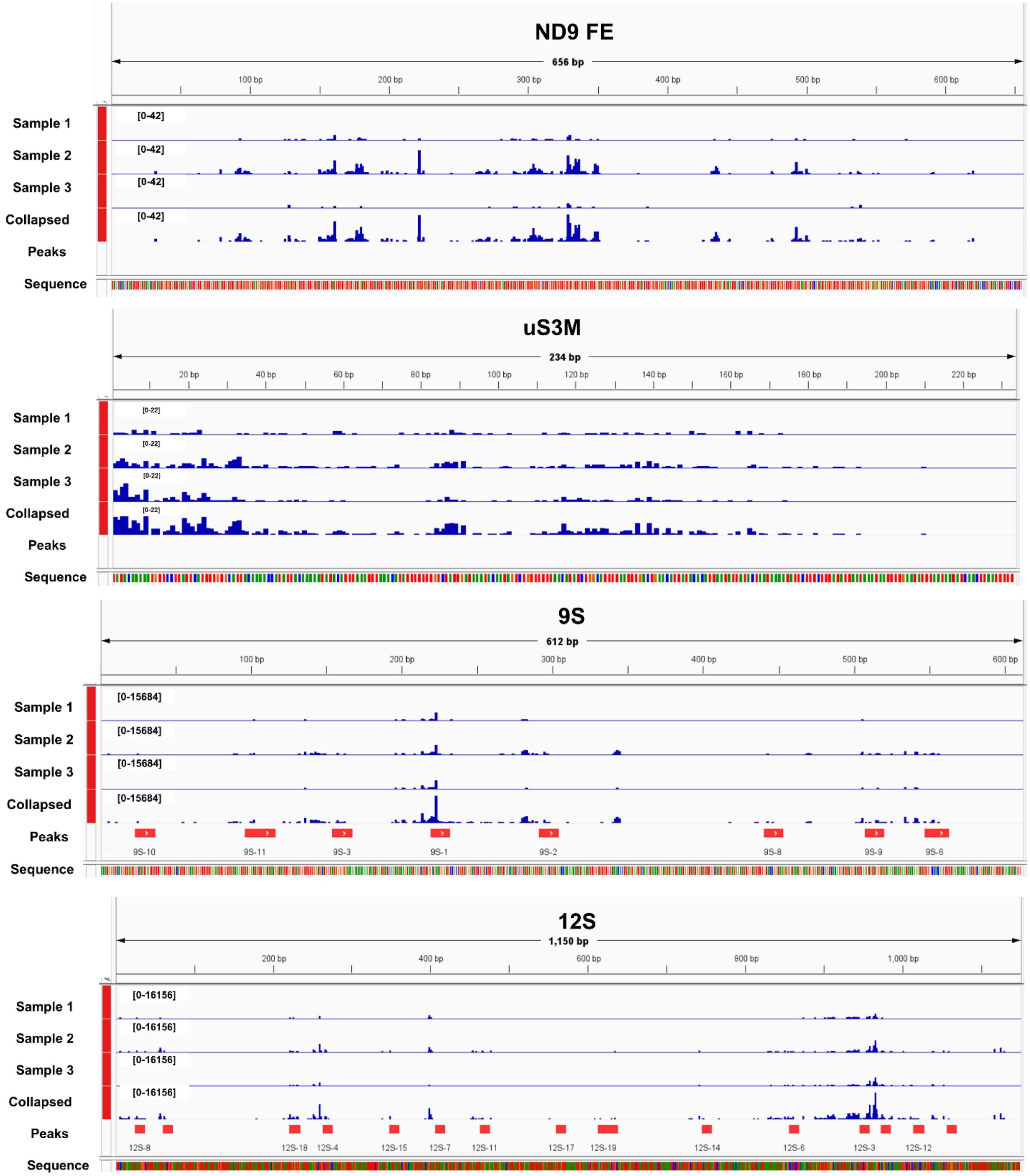

Figure S6: eCLAP analysis of KRBP72 on mitochondrial RNA (pre-edited, fully edited, and never edited). KRBP72 binding sites are shown in blue bars and high confidence peaks are indicated by the red bars. Three biological replicates (Samples 1-3) and combined binding sites (Collapsed) are shown.

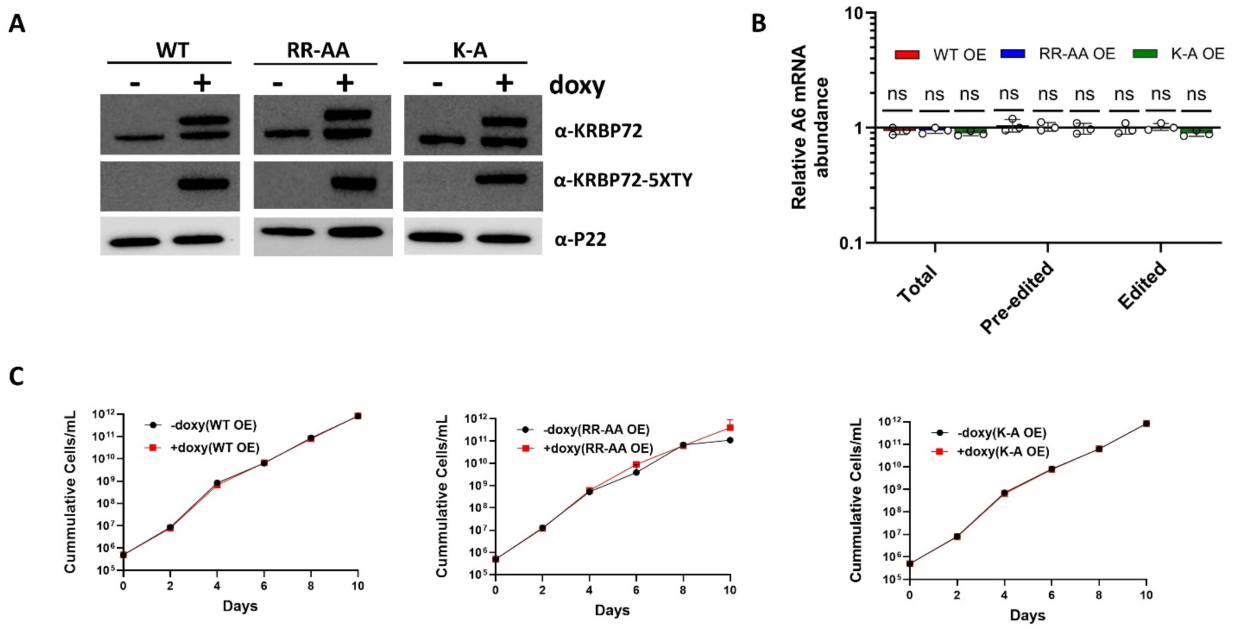

Figure S7: Effect of overexpression of WT, RR-AA mutant, and K-A mutant KRBP72 in PCF *T. brucei* cells. **(A)** Western blot showing the expression of both endogenous KRBP72 and recoded WT and mutant proteins expression level. Anti-KRBP72 detects endogenous and overexpressed proteins, while anti-TY detects only the overexpressed protein. P22 serves as a loading control. **(B)** RNA was isolated on day 3 post-induction. A6 mRNA levels were quantified by qRT-PCR using primer sets specific for total, pre-edited, and fully edited RNAs. Relative RNA abundance represents levels in induced (+doxy) cells compared to levels in uninduced (-doxy) cells, normalized to 18S rRNA levels. Three biological replicates were performed, each with three technical replicates. Significance was evaluated using Student's t-test. ns, not significant **(C)** Cell growth was monitored for 10 days in triplicate for both uninduced (-doxy) and induced cells (+doxy) for each of the three the cell lines.

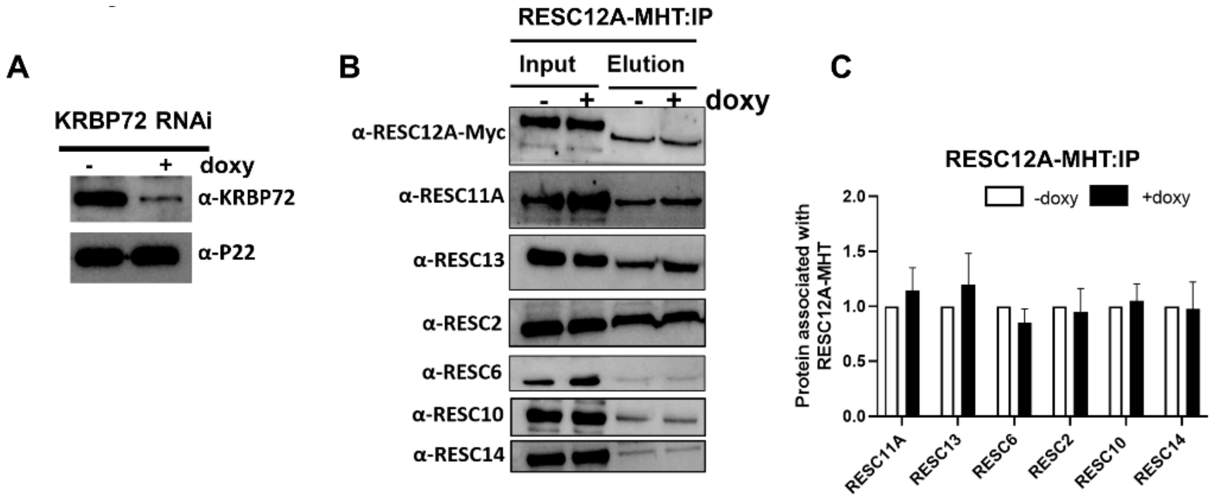

Figure S8: PCF *T. brucei* cells containing both the KRBP72 RNAi construct and the RESC12A-MHT construct were utilized to examine the proteins associated with the RESC subcomplex during KRBP72 depletion. Cells were cultured for 3 days in the absence or presence of doxy. **(A)** KRBP72 RNAi was confirmed by western blot using KRBP72 antibody; P22 served as a loading control. **(B)** RESC12A-MHT was pulled down using IgG beads; bound proteins were eluted through TEV protease cleavage. Elutions were subsequently examined by western blotting with anti-RESC protein antibodies. Two biological replicate experiments were conducted. Protein levels were standardized to the amount of RESC12A for that immunoprecipitation (IP). **(C)** The level of proteins associated with RESC12A was then determined by comparing the normalized protein levels from the +doxy IP to that of the −doxy IP (set to 100%). Bar graphs depict the average and standard deviation of two biological replicates.

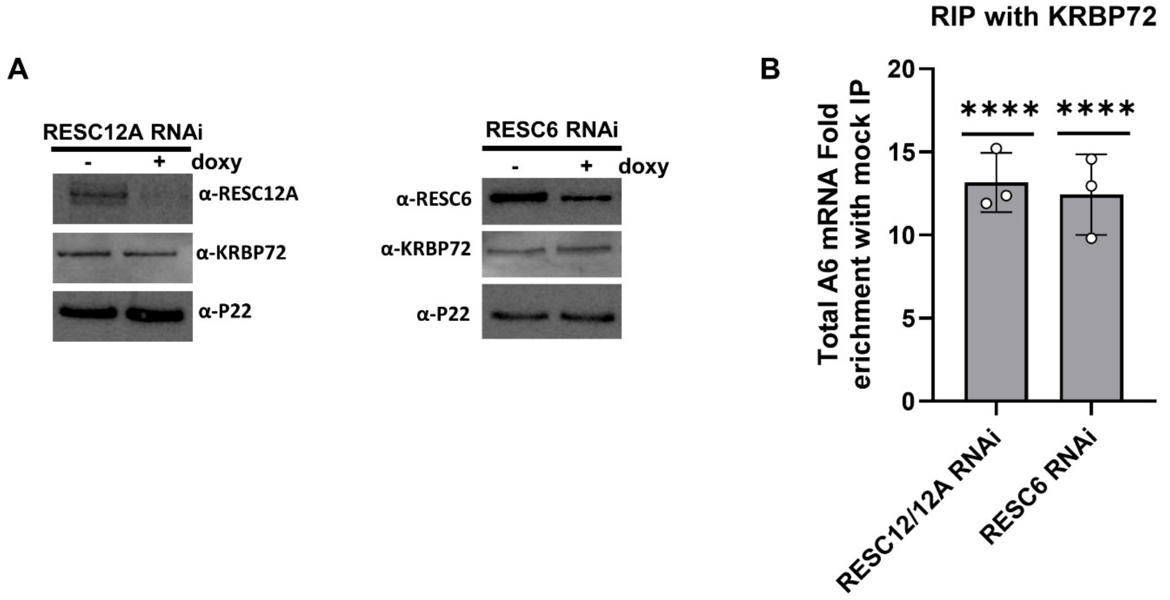

Figure S9: Controls for KRBP72 RIP experiments. **(A)** RESC6 and RESC12A knockdown. RNAi cells were grown for 3 days either induced (+doxy) or uninduced (-doxy). RESC6 and RESC12A RNAi was confirmed by using RESC6 and RESC12A antibodies, respectively. P22 served as a loading control. KRBP72 abundance does not change in either of the RNAi cell lines compared to control. **(B)** Enrichment of total A6 mRNA in KRBP72 RIP compared to beads alone. RNA levels were standardized against 18S rRNA, and numbers represent the three biological replicates, each with three technical replicates. Significance was evaluated using Student's t-test. *** P < 0.0001.

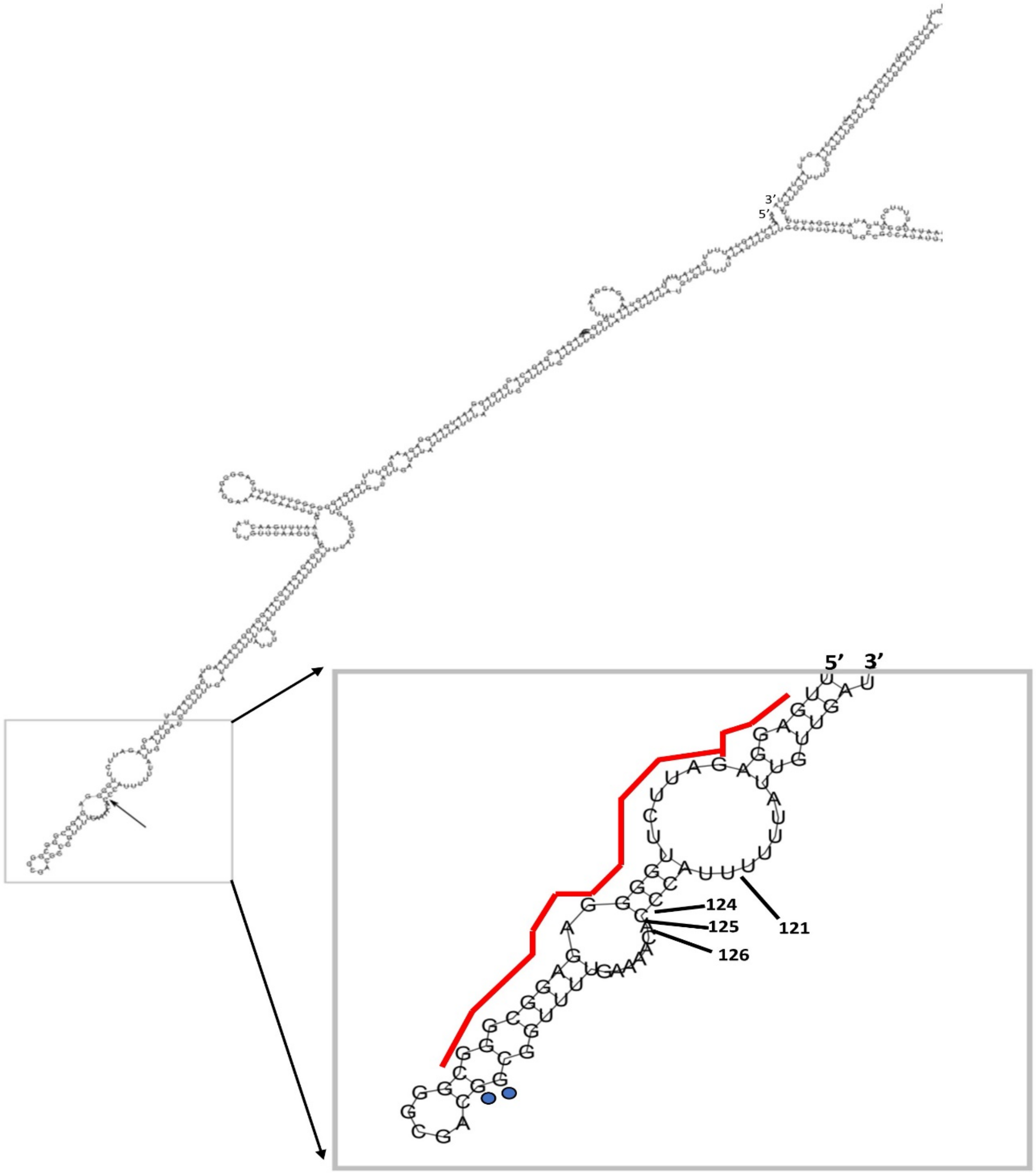

Figure S10: Structure prediction of A6 partially edited mRNA (fully edited 3’ of ES 124 and pre-edited 5’ of this site). Predictions were carried out using the Vienna RNA cofold function at a temperature of 27°C. The zoomed-in region within the rectangle highlights the area under study in this manuscript with editing sites shown to be prominent EJES in KRBP72 RNAi cells denoted by numbers. Red lines above the structure indicate two closely spaced high-confidence peaks, while blue dots mark two sites with strong binding peaks.
